# Fas (CD95) expression in myeloid cells promotes obesity-induced muscle insulin resistance

**DOI:** 10.1002/emmm.201302962

**Published:** 2013-11-06

**Authors:** Stephan Wueest, Rouven Mueller, Matthias Blüher, Flurin Item, Annie S H Chin, Michael S F Wiedemann, Hitoshi Takizawa, Larisa Kovtonyuk, Alexander V Chervonsky, Eugen J Schoenle, Markus G Manz, Daniel Konrad

**Affiliations:** 1 Division of Pediatric Endocrinology and Diabetology, University Children's HospitalZurich, Switzerland; 2 Children's Research Centre, University Children's HospitalZurich, Switzerland; 3 Division of Hematology, University Hospital ZurichZurich, Switzerland; 4 University of Leipzig, Department of MedicineLeipzig, Germany; 5 Zurich Centre for Integrative Human Physiology, University of ZurichZurich, Switzerland; 6 Department of Pathology, University of ChicagoChicago, IL, USA

**Keywords:** diabetes mellitus, insulin resistance, obesity

## Abstract

Low-grade inflammation in adipose tissue and liver has been implicated in obesity-associated insulin resistance and type 2 diabetes. Yet, the contribution of inflammatory cells to the pathogenesis of skeletal muscle insulin resistance remains elusive. In a large cohort of obese human individuals, blood monocyte *Fas* (*CD95*) expression correlated with systemic and skeletal muscle insulin resistance. To test a causal role for myeloid cell Fas expression in the development of skeletal muscle insulin resistance, we generated myeloid/haematopoietic cell-specific Fas-depleted mice. Myeloid/haematopoietic Fas deficiency prevented the development of glucose intolerance in high fat-fed mice, in *ob*/*ob* mice, and in mice acutely challenged by LPS. *In vivo*, *ex vivo* and *in vitro* studies demonstrated preservation of muscle insulin responsiveness with no effect on adipose tissue or liver. Studies using neutralizing antibodies demonstrated a role for TNFα as mediator between myeloid Fas and skeletal muscle insulin resistance, supported by significant correlations between monocyte *Fas* expression and circulating TNFα in humans. In conclusion, our results demonstrate an unanticipated crosstalk between myeloid cells and skeletal muscle in the development of obesity-associated insulin resistance.

## Introduction

Obese people are prone to type 2 diabetes, arising from relative insulin deficiency combined with insulin resistance. The latter is defined as impaired ability of insulin to promote glucose uptake into skeletal muscle and adipose tissue as well as to inhibit hepatic glucose production. Since skeletal muscle is the predominant site of insulin-mediated glucose disposal in the postprandial state (DeFronzo & Tripathy, 2009), development of muscular insulin resistance will crucially impact on glucose homeostasis. In fact, skeletal muscle insulin resistance is an early metabolic alteration that frequently precedes type 2 diabetes (DeFronzo & Tripathy, 2009). In obesity, low-grade inflammation frequently develops systemically and in different organs such as adipose tissue, liver or pancreatic islets, and is thought to play a causative role in the pathogenesis of insulin resistance and type 2 diabetes (Donath & Shoelson, 2011; Lazar, 2005; Weisberg *et al,* 2003; Xu *et al,* 2003; Yuan *et al,* 2001). It is characterized by elevated levels of pro-inflammatory cytokines, and by infiltration of these tissues with macrophages and other immune cells (Nishimura *et al,* 2009; Weisberg *et al,* 2003; Winer *et al,* 2009, 2011; Xu *et al,* 2003). Moreover, although macrophage accumulation was mainly studied in adipose tissue, skeletal muscle may also be infiltrated, a process proposed to contribute to high fat diet (HFD) induced insulin resistance (Olefsky & Glass, 2010). However, the contribution of inflammatory cells including monocytes and macrophages to skeletal muscle insulin resistance remains poorly understood (Mauer *et al,* 2010; Samokhvalov *et al,* 2009).

Fas–FasL interaction is regarded as an important action in immune responses. Besides its well-characterized role in the induction of programmed cell death (apoptosis), there is evidence that Fas activation may induce non-apoptotic signalling pathways such as inflammatory cascades (Guo *et al,* 2005; Peter *et al,* 2007; Schumann *et al,* 2007; Wajant *et al,* 2003). Accordingly, activation of the Fas signalling pathway was shown to induce secretion of pro-inflammatory cytokines like IL-1α, IL-1β, IL-6, IL-8 (keratinocyte chemoattractant (KC)), MCP-1 and TNFα in different cell types such as adipocytes and monocytes (Faouzi *et al,* 2001; Farley *et al,* 2008; Imamura *et al,* 2004; Miwa *et al,* 1998; Park *et al,* 2003; Schaub *et al,* 2003; Wang *et al,* 2010; Wueest *et al,* 2010b), rendering it a potential key component of the inflammatory response. We previously found that Fas ablation specifically in adipocytes reduced adipose tissue inflammation and hepatic insulin resistance in high fat-fed mice, highlighting a role for Fas in the disturbed adipocyte–hepatocyte communication observed in obesity (Wueest *et al,* 2010b).

In the present study, we hypothesized that myeloid cell-expressed Fas impacts on inflammatory pathways and, consequently, on glucose homeostasis. To test this hypothesis, monocytic Fas expression was analysed in a large cohort of obese human individuals (*n* = 246) and correlated with the parameters insulin sensitivity and glucose metabolism. In addition, glucose metabolism was determined in standard chow-fed, HFD-fed or LPS-injected control and myeloid cell-specific Fas-depleted mice on a C57BL6/J or *ob*/*ob* background. Our results propose a thus far unknown Fas-mediated crosstalk between myeloid cells and skeletal muscle contributing to the development of obesity-associated insulin resistance.

## Results

### Fas expression in circulating monocytes correlates with insulin resistance and type 2 diabetes in obese patients

**Figure 1:**
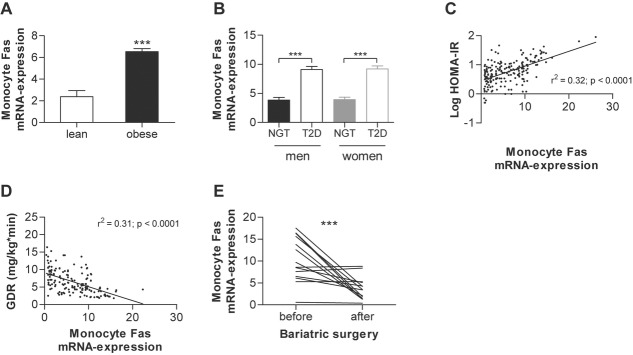
Fas expression in circulating monocytes correlates negatively with insulin sensitivity in obese patients A  Monocytes were isolated from whole human blood samples of lean and obese subjects and Fas mRNA expression was measured normalized to HPRT. *n* = 16–247. **p = 0.005 (Student's *t*-test). Error bars represent SEM. B  Monocytes were isolated from whole human blood samples and Fas mRNA expression was measured normalized to HPRT. ***p < 0.0001 (Student's *t*-test). NGT: normal glucose tolerance (men *n* = 61; women *n* = 70); T2D: type 2 diabetes (men *n* = 57; women *n* = 58). Error bars represent SEM. C,D  Monocyte Fas mRNA expression was determined as described above and correlated to HOMA-IR (C) and glucose disposal rate (GDR) (D). *n* = 200 (C) and *n* = 163 (D). Error bars represent SEM. E  Monocyte Fas mRNA expression was determined in obese patients before and 6 months after bariatric surgery (gastric sleeve resection; *n* = 14), ***p = 0.0002 (Student's *t*-test).

To unravel whether obesity has an impact on myeloid *Fas* expression, mRNA levels were determined in circulating monocytes of lean and obese human subjects (body mass index (BMI): 21.4 ± 0.5 kg/m^2^ in lean vs. 45.9 ± 1.1 kg/m^2^ in obese subjects, *p* < 0.0001). Intriguingly, *Fas*expression was significantly increased in obese compared to lean subjects (Fig 1A). To further determine if Fas expression in circulating monocytes signifies a more metabolically morbid sub-phenotype of human obesity, monocytic *Fas* mRNA expression was analysed in obese humans with either normal glucose tolerance (NGT; *n* = 131) or with type 2 diabetes (*n* = 115). Basic clinical characteristics of these subjects are provided in Table 1. *Fas* expression was not different between males and females, but higher in monocytes of obese persons with type 2 diabetes compared to obese, normal glucose tolerant subjects (Fig 1B). Similar to Fas*, Fas ligand (FasL)* expression was increased in obese persons with type 2 diabetes (supplementary Fig 1). Strikingly, *Fas* expression in human circulating monocytes positively correlated with HOMA-IR (Fig 1C), a measure of systemic insulin resistance. To gain more insight into potential mechanisms linking monocytic Fas expression and insulin resistance, hyperinsulinaemic-euglycaemic clamp studies were performed. Fas mRNA in circulating monocytes correlated negatively with glucose disposal rate (GDR), a measure mainly reflecting skeletal muscle insulin sensitivity (Fig 1D). Complementary to the cross-sectional study, surgery-induced weight loss, which resulted in significantly improved insulin sensitivity (supplementary Fig 2), also resulted in a significant decline in monocyte *Fas* mRNA expression (Fig 1E). Clinical characteristics of these subjects are provided in Table 1. Importantly, HOMA-IR correlated with monocyte *Fas* expression at baseline in the bariatric surgery group (supplementary Fig 3) and changes in HOMA-IR 6 months after bariatric surgery significantly correlated with changes in monocyte *Fas* mRNA expression, even after adjustment for changes in BMI (*r* = 0.14, adjusted *p* = 0.042). Jointly, these cross-sectional and longitudinal associations between monocyte-expressed *Fas* and insulin resistance inspired us to hypothesize that monocyte Fas plays a causal role in obesity-associated skeletal muscle insulin resistance. To test this hypothesis, we generated myeloid-specific Fas-knockout mice.

**Table 1:** Basic clinical characteristics of patients

	Cross-sectional study		Bariatric surgery intervention	
	NGT	T2D	Baseline	6 Months post
Age	41.3 ± 14.6	51.9 ± 13	41.9 ± 13.3	
Gender (M/F)	61/70	57/58	4/10	
BMI (kg/m^2^)	40.2 ± 11.7	47.7 ± 10.8	54.1 ± 8.1	36.3 ± 7.3^b^
Body fat (%)	37.2 ± 11.9	42.1 ± 10.2	56.1 ± 8.3	38.5 ± 7.2^b^
HbA1c (%)	5.7 ± 0.3	7.1 ± 1.0^a^	6.6 ± 0.9	5.8 ± 0.35^b^
FPG (mmol/L)	5.5 ± 0.6	7.2 ± 1.5^a^	6.1 ± 0.6	5.7 ± 0.3^b^
FPI (pmol/L)	200 ± 246	266 ± 233	382 ± 164	183 ± 72^b^
HOMA-IR	7.0 ± 0.9	12.3 ± 2.2^a^	14.9 ± 0.6	6.7 ± 0.1
Triglycerides (mmol/L)	2.0 ± 1.1	2.48 ± 1.3^a^	2.3 ± 0.5	1.75 ± 0.3^b^
HDL-cholesterol (mmol/L)	1.24 ± 0.3	1.15 ± 0.3	0.91 ± 0.18	1.23 ± 0.2^b^
hsCrP (mg/L)	0.85 ± 1.2	1.1 ± 0.9	4.36 ± 0.7	2.76 ± 0.7^b^

a *p *<* *0.05 for comparisons between NGT and T2D.

b *p *< 0.05 for comparisons between 6 months post bariatric surgery and baseline.

### Myeloid cell-specific Fas deletion protects from HFD-induced muscle insulin resistance

**Figure 2:**
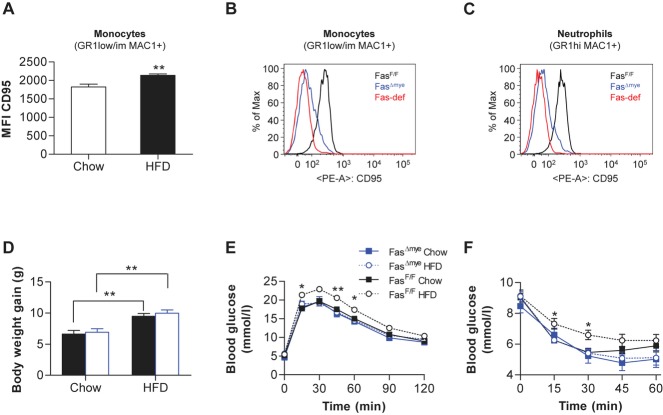
Fas^Δmye^ mice are protected from HFD-induced glucose intolerance. A  Flow cytometric analysis of peripheral blood leukocytes of chow- and HFD-fed mice. Monocytes (GR1low/im MAC1+) were stained with respective antibodies. Bar graphs show mean fluorescence intensity (MFI) of Fas (CD95) of live monocytes. *n* = 6–8, **p = 0.005 (Student's *t*-test). Error bars represent SEM. B,C  Flow cytometric analysis of circulating monocytes (B) and neutrophils (C) of Fas-deficient, Fas^F/F^ and Fas^Δmye^ mice. Cells were stained with respective antibodies and Fas fluorescence was measured. D  Body weight change during 6 weeks of chow- or HFD-fed in Fas^F/F^ and Fas^Δmye^ mice. *n* = 7–20. **p = 0.002 (Fas^F/F^ chow *vs*. HFD); **p = 0.001 (Fas^Δmye^ chow *vs*. HFD; Student's *t*-test). Error bars represent SEM. E  Intra-peritoneal glucose-tolerance test in chow- and HFD-fed Fas^F/F^ and Fas^Δmye^ mice at 12 weeks of age. *n* = 6–10. *p = 0.049 and 0.035 after 15 and 60 min, respectively; **p = 0.008 (ANOVA). All error bars represent SEM. F  Intra-peritoneal insulin-tolerance test in chow- and HFD-fed Fas^F/F^ and Fas^Δmye^ mice at 12 weeks of age. *n* = 6–10. *p = 0.034 (15 min), *p = 0.012 (30 min; ANOVA). All error bars represent SEM.

In wild-type mice, HFD-induced obesity was associated with elevated Fas levels in circulating monocytes as determined by flow cytometric analysis (Fig 2A and supplementary Fig 4). In contrast, HFD did neither increase Fas levels in B- and T-lymphocytes nor in neutrophils (supplementary Fig 5). In order to further assess a role for myeloid-expressed Fas in the development of obesity-associated insulin resistance, myeloid-specific Fas-knockout mice (Fas^f/f^, LysM-Cre^+/−^; Fas^Δmye^) were generated using the cre-lox system (Clausen *et al,* 1999; Olefsky & Glass, 2010). LysM-Cre mice allow specific and highly efficient deletion of *loxP*-flanked target genes in myeloid cells, *i.e*. macrophages, monocytes, neutrophil granulocytes and partially dendritic cells (Clausen *et al,* 1999). As controls, littermate mice with floxed Fas but absent Cre-recombinase (Cre) expression were used (Fas^f/f^, LysM-Cre^−/−^; Fas^F/F^). Flow cytometric analysis revealed diminished Fas protein levels in circulating monocytes and neutrophils of Fas^Δmye^ mice (Fig 2B and C). However, no reduction was observed in non-myeloid B- and T-lymphocytes (supplementary Fig 6A). Western blot analysis confirmed reduced Fas protein content in isolated macrophages of Fas^Δmye^ mice whereas it was unchanged in white adipose tissue, liver and other tissues (supplementary Fig 6B). To investigate the functional significance of myeloid-specific Fas-deletion, Fas^Δmye^ mice were fed a standard chow or HFD for 6 weeks. Total body weight gain was similar in Fas^Δmye^ and their Cre-negative littermates under both diets (Fig 2D), and no significant differences were observed in either fat pad weights or adipocyte size distribution (supplementary Fig 7A–C). Moreover, *FasL* expression in myeloid cells was similar between both genotypes upon HFD (supplementary Fig 8). Strikingly, whereas 6 weeks of HFD impaired glucose and insulin tolerance tests in Fas^F/F^ compared to chow-fed mice, Fas^Δmye^ mice showed no deterioration in glucose metabolism (Fig 2E and F). In addition, fasting blood glucose levels were significantly lower in HFD-fed Fas^Δmye^ compared to Fas^F/F^ littermates, whereas insulin, free fatty acid (FFA) and triglyceride (TG) levels as well as circulating adiponectin and leptin levels did not differ significantly between the two genotypes (Table 2). The protective effect against HFD-induced glucose and insulin intolerance by myeloid cell-specific Fas deletion could not be attributed to differences in food intake, locomotion or fuel utilization (respiratory quotient, RQ; supplementary Fig 9A–C).

**Table 2:** Phenotypic characteristics of HFD-fed Fas^F/F^ and Fas^Δmye^ mice

	Fas^F/F^	Fas^Δmye^
Body weight (g)	28.8 ± 0.8	28.2 ± 0.7
Blood glucose (mmol/L)	9.5 ± 0.5	8.3 ± 0.3*
Insulin (pmol/L)	146.6 ± 19.9	120.5 ± 31.4
FFA (mmol/L)	0.77 ± 0.09	0.77 ± 0.08
TG (mg/dl)	117.7 ± 29.7	106.9 ± 13.9
Adiponectin (μg/ml)	41.4 ± 6.1	31.1 ± 4.6
Leptin (pg/ml)	48.1 ± 11.5	44.7 ± 9.8
IL-6 (pg/ml)	4.1 ± 0.7	2.6 ± 1.3
MCP-1 (pg/ml)	6.8 ± 0.8	6.4 ± 0.5
IL-10 (pg/ml)	n.d.	n.d.
LPS (EU/ml)	8.6 ± 1.1	8.3 ± 1.2

n.d. = not detectable.

Mice were fasted for 8 h. Results are the means ± SEM of 5–15 mice.

* *p* = 0.049 (Student's *t*-test).

**Figure 3:**
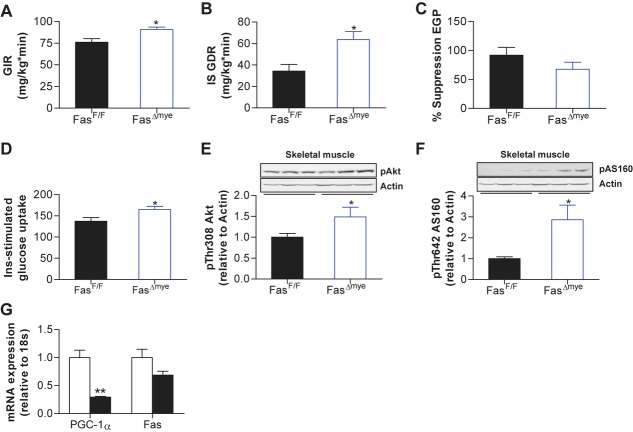
Fas^Δmye^ mice are protected from HFD-induced muscle insulin resistance. A–C  Glucose infusion rate (GIR), insulin-stimulated glucose disposal rate (IS GDR) and% suppression of endogenous glucose production (EGP) during hyperinsulinaemic-euglycaemic clamps, *n* = 4–5, *p = 0.026 (A) and *p = 0.018 (B; Student's *t*-test). Error bars represent SEM. D  Insulin-stimulated glucose uptake into isolated soleus muscle of HFD-fed Fas^F/F^ and Fas^Δmye^ mice relative to basal uptake. *n* = 4–6, *p = 0.039 (Student's *t*-test). Error bars represent SEM. E,F  Representative Western blots of total muscle lysates of Fas^F/F^ and Fas^Δmye^ mice. Graphs show results of 6–10 (E) and 7–8 (F) mice. *p = 0.036 (E) and *p = 0.031 (F) (Student's *t*-test). Error bars represent SEM. G  mRNA expression of respective genes in skeletal muscle of chow (white bars) and HFD-fed (black bars) mice. *n* = 4. **p = 0.002 (Student's*t*-test). Error bars represent SEM.

To better elucidate the metabolic-endocrine phenotype of HFD-fed Fas^Δmye^ mice, hyperinsulinaemic-euglycaemic clamp studies were performed. A significantly increased glucose infusion rate in HFD-fed Fas^Δmye^ compared to Fas^F/F^ mice was noted, consistent with improved whole-body insulin sensitivity (see Fig 3A and supplementary Fig 10A–C for detailed time courses). Importantly, insulin-stimulated glucose disposal rate was higher in Fas^Δmye^ compared to Fas^F/F^ littermates suggesting improved skeletal muscle insulin sensitivity (Saberi *et al,* 2009b; Fig 3B). Remarkably, such protective effect against insulin resistance seemed to be specific to skeletal muscle since insulin-mediated inhibition of endogenous (mainly reflecting hepatic) glucose production was not different between Fas^F/F^ and Fas^Δmye^ mice (Fig 3C) as was insulin-mediated decrease of circulating FFA levels (supplementary Fig 11). Consistent with clamp studies, insulin-stimulated glucose uptake into isolated soleus muscle was significantly increased in HFD-fed Fas^Δmye^ compared to Fas^F/F^ littermates (Fig 3D), corresponding to increased insulin-induced phosphorylation of Akt and AS160 in skeletal muscle of myeloid-specific Fas knockout mice (Fig 3E and F). In contrast, no significant differences in insulin-stimulated Akt phosphorylation were observed in white adipose tissue or liver of high fat-fed Fas^Δmye^ and Fas^F/F^ mice (supplementary Fig 12A and B). Moreover, mRNA expression of cytokines and macrophage markers in adipose tissue and liver were comparable in both groups suggesting that myeloid cell-expressed Fas does not have a major impact on adipose tissue and/or liver inflammation (supplementary Fig 12C and D). In addition, flow cytometric analysis revealed similar immune cell infiltration and macrophage polarization in adipose tissue and livers of HFD-fed control and myeloid-specific Fas knockout mice (supplementary Fig 12E–G).

To further characterize skeletal muscle from Fas^Δmye^ mice, mRNA expression of cytokines as well as markers of β-oxidation and lipogenesis were measured, and were found to be expressed at similar levels in HFD-fed knockout and control mice (supplementary Fig 13). Importantly, there was neither a difference in inflammatory cell marker expression nor in immune cell infiltration and macrophage polarization in skeletal muscle between Fas^F/F^ and Fas^Δmye^ littermates as determined by real-time RT-PCR and flow cytometry (supplementary Fig 13A and 13B). Moreover, HFD did not induce *Fas* expression in skeletal muscle of C57BL6/J mice (Fig 3G) and Fas protein levels could neither be detected in skeletal muscle of HFD-fed Fas^F/F^ nor Fas^Δmye^ mice, confirming previous findings (Wueest *et al,* 2010b). Hence, improved skeletal muscle insulin sensitivity in myeloid-specific Fas knockout mice is likely driven by an endocrine process, rather than a paracrine effect within skeletal muscle itself.

To further confirm the causal role of myeloid Fas in obesity-associated skeletal muscle insulin resistance, we generated mice deficient of Fas in all haematopoietic-derived cells using adoptive bone marrow (BM) transfer. Wild-type C57BL6/J mice receiving a BM transplant from total body Fas-deficient (BM Fas-def) mice showed a marked reduction in Fas protein content in myeloid cells compared to BM WT mice (supplementary Fig 14). Confirming our results from myeloid-specific Fas knockout mice generated with the cre-lox technique, BM Fas-def chimeras were protected from HFD-induced skeletal muscle insulin resistance (supplementary Fig 15). Phenotypic characteristics of these mice are reported in supplementary Table S1.

### Fas depletion in haematopoietic cells of ob/ob mice improves skeletal muscle insulin sensitivity

**Figure 4:**
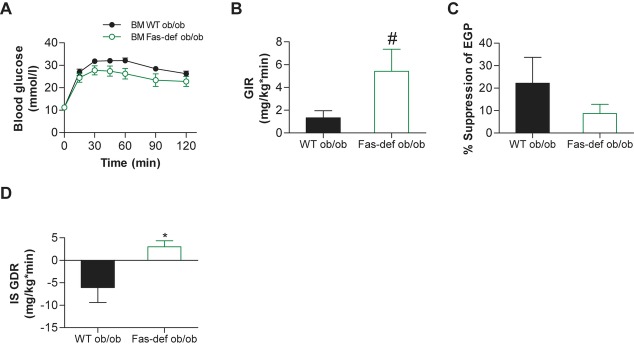
ob/ob Bone marrow chimeras with Fas-depleted myeloid cells are protected from skeletal muscle insulin resistance. A–D  (A) Intra-peritoneal glucose-tolerance test and hyperinsulinaemic-euglycaemic clamp studies (B–D) with glucose infusion rate (GIR), insulin-inhibited endogenous glucose production (EGP) and insulin-stimulated glucose disposal rate (IS GDR) were performed in ob/ob BM WT and ob/ob BM Fas-def mice. *n* = 5–6, ^#^*p* = 0.09 (Student's *t*-test), *p = 0.025 (Student's *t*-test). All error bars represent SEM.

To further unravel a potential role of myeloid-expressed Fas in modulating skeletal muscle insulin resistance and to rule out any specific effects of the HFD, Fas was depleted in myeloid/haematopoietic cells of the leptin-deficient (*ob*/*ob*) mouse using adoptive BM transfer. Eight weeks after transplantation, glucose tolerance was improved in chimeric Fas-def *ob*/*ob* mice (Fig 4A). Moreover, glucose infusion rate (GIR) during hyperinsulinaemic-euglycaemic clamp studies was higher in Fas-def *ob*/*ob* compared to WT *ob*/*ob* mice (see Fig 4B for steady-state glucose infusion rates and supplementary Fig 16 for detailed time courses). Importantly, observed differences in GIR between the two groups were not due to differences in hepatic insulin sensitivity (Fig 4C), but rather to significantly improved skeletal muscle insulin sensitivity in Fas-def ob/ob mice (Fig 4D).

Jointly, these results suggest that depletion of Fas in myeloid/haematopoietic cells as demonstrated either by lineage-specific elimination or by total BM transplantation protects mice from obesity-induced skeletal muscle insulin resistance, without affecting hepatic or adipose tissue insulin sensitivity or HFD-induced weight gain.

### Fas mediates myeloid-muscle cell communication leading to myocellular insulin resistance

**Figure 5:**
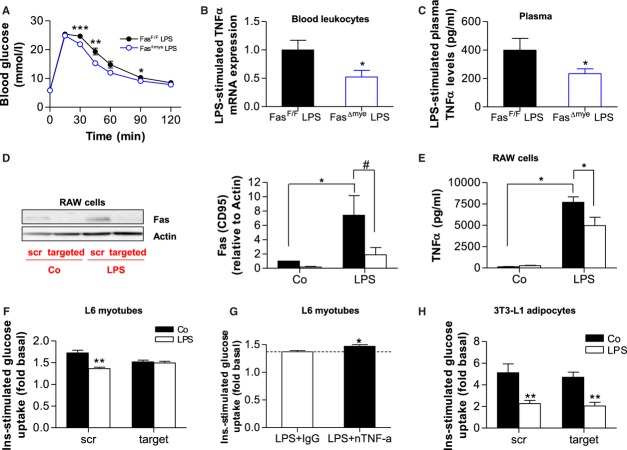
Fas mediates myeloid-muscle cell communication leading to myocellular insulin resistance. A  Intra-peritoneal glucose-tolerance test in Fas^F/F^ and Fas^Δmye^ mice. LPS (1 mg/kg BW) was injected 45 min prior to glucose tolerance test. *n* = 7, *p = 0.022, **p = 0.005, ***p = 0.0007 (Student's *t*-test). Error bars represent SEM. B,C  Leukocyte-mRNA expression of TNFα (*n* = 6–7; B) and circulating plasma TNFα levels (*n* = 8–13; C) of Fas^F/F^ (black bars) and Fas^Δmye^(blue bars) mice 60 min upon intraperitoneal injection of 1 mg/kg BW LPS. *p = 0.049 (Student's *t*-test). Error bars represent SEM. D  Representative Western blot and quantitative analysis of total lysates of RAW cells treated with scrambled (scr, black bars) or targeted (white bars) Fas siRNA for 48 h and subsequently stimulated with or without LPS (100 ng/ml for 6 h). *n* = 4–5, *p = 0.01 (Mann–Whitney test, scr Co *vs*. scr LPS) and ^#^*p* = 0.07 Student's *t*-test, scr LPS *vs*. target LPS). Error bars represent SEM. E  RAW cells were treated with scrambled (scr, black bars) or target (white bars) Fas siRNA for 48 h. Thereafter, cells were stimulated with or without 100 ng/ml LPS for 6 h and cytokine release was measured. *n* = 6. *p = 0.047 (scr Co *vs*. scr LPS) and *p = 0.042 (scr LPS *vs*. target LPS; Student's *t*-test). Error bars represent SEM. F  L6 myotubes were incubated overnight with conditioned media from RAW cells treated as mentioned above and insulin-stimulated glucose uptake was measured. *n* = 4–7, **p = 0.002 (scr Co *vs*. scr LPS; Student's *t*-test). Error bars represent SEM. G  L6 myotubes were incubated overnight with conditioned media from LPS-stimulated RAW cells in the presence of IgG control (open bar) or nTNFα (black bar) antibody and insulin-stimulated glucose uptake was measured. Results are expressed relative to respective unstimulated values. *n* = 5, *p = 0.041. Error bars represent SEM. H  Mature 3T3-L1 adipocytes were incubated overnight with conditioned media from RAW cells treated as mentioned above and insulin-stimulated glucose uptake was measured. *n* = 4, **p = 0.016 (scr) and **p = 0.04 (target; Student's *t*-test). Error bars represent SEM.

Lipopolysaccharides (LPS) injections were previously shown to induce insulin resistance in mice (Arkan *et al,* 2005; Ling *et al,* 1994). In such treated animals, inflammation is mimicked in the absence of chronic caloric surplus and tissue infiltration with inflammatory cells. Moreover, the relevance of such approach is further strengthened by the finding that plasma LPS levels are elevated in HFD-fed mice due to increased absorption of LPS from the gut and, thus, LPS was postulated to initiate obesity and insulin resistance (Cani *et al,* 2007; Ley *et al,* 2006). In order to explore the notion whether myeloid cell-specific Fas deletion protects against LPS-induced insulin resistance, LPS was injected intraperitoneally (1 mg/kg body weight). As depicted in Fig 5A, glucose tolerance was impaired in Fas^F/F^ mice, whereas Fas^Δmye^ littermates were partly protected from LPS-induced deterioration of glucose metabolism. Of note, insulin levels after LPS injection were similar in both groups (Fas^F/F^ 131.6 ± 22.6 pmol/L *vs*. Fas^Δmye^ 138.0 ± 18.9 pmol/L).

LPS was previously demonstrated to increase circulating TNFα and IL-6 levels in mice (McIlwain *et al,* 2012). Moreover, interruption of Fas–FasL signalling suppressed cytokine expression induced by LPS in mouse macrophages (Ma *et al,* 2004). As shown in Fig 5B, LPS-stimulated mRNA expression of TNFα but not IL-6 (data not depicted: 1.0 ± 0.28 *vs*. 0.74 ± 0.28; *p* = 0.51) was significantly reduced in circulating immune cells isolated from Fas^Δmye^ mice. Moreover, LPS-induced circulating TNFα levels were significantly lower in Fas^Δmye^ compared to Fas^F/F^ mice (Fig 5C). Similar to findings presented in mice, LPS increased Fas expression in RAW 264.7 cells (a murine myeloid cell line), an effect that was greatly blunted after siRNA-mediated depletion of Fas expression (Fig 5D). Concomitantly, Fas down-regulation in RAW cells significantly decreased LPS-induced release of TNFα into culture medium (Fig 5E), suggesting a role for Fas in LPS-induced TNFα secretion. In contrast to Fas, its ligand (FasL) is not induced in RAW cells after LPS stimulation (supplementary Fig 17).

To investigate a potential role of Fas in mediating myeloid-muscle inter-cellular communication, the effect of conditioned medium harvested from RAW cells was tested on insulin signalling and metabolism in L6 myotubes, mature 3T3-L1 adipocytes or HepG2 hepatocytes. Conditioned medium from RAW cells over-expressing Fas (in response to LPS stimulation) decreased insulin-stimulated glucose uptake into L6 myotubes (Fig 5F), potentially recapitulating the link between obesity-induced monocyte Fas over-expression and muscular insulin resistance (Fig 1). Fas' causal role in the process was supported by demonstrating a reversal of this effect when Fas-depleted RAW cells were used (Fig 5F). To unravel a possible role for reduced LPS-stimulated TNFα release from Fas-depleted RAW cells, insulin-stimulated glucose uptake into L6 myotubes was performed in the presence of neutralizing TNFα antibody. The inhibitory effect of LPS-treated RAW cell media on insulin-stimulated glucose uptake was blunted after TNFα neutralization (Fig 5G) suggesting a TNFα-dependent effect of conditioned media on insulin resistance. Importantly, Fas-depletion in myeloid cells had no protective effect on conditioned media-induced insulin resistance in mature 3T3-L1 adipocytes (Fig 5H) or in HepG2 hepatocytes (supplementary Fig 18).

**Figure 6:**
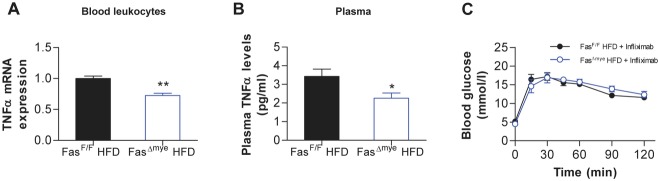
Reduced circulating TNFα levels in Fas^Δmye^ mice. A,B  Leukocyte-mRNA expression of TNFα (*n* = 3–5; A) and circulating plasma TNFα levels (*n* = 12–15; B) of HFD-fed Fas^F/F^ (black bars) and Fas^Δmye^ (blue bars) mice. *p = 0.033, **p = 0.003 (Student's *t*-test). Error bars represent SEM. C  Intra-peritoneal glucose tolerance test in HFD-fed Fas^F/F^ and Fas^Δmye^mice pre-treated with the TNFα neutralizing antibody infliximab. *n* = 4. All error bars represent SEM.

To further unravel a causative role for TNFα in mediating obesity-induced myeloid Fas-dependent muscle insulin resistance, its expression and plasma levels were analysed. As shown in Fig 6A, *TNF*α expression was significantly reduced in circulating immune cells isolated from HFD-fed Fas^Δmye^ compared to Fas^F/F^ mice. Moreover, circulating TNFα concentrations were significantly reduced in HFD-fed Fas^Δmye^ mice (Fig 6B), while plasma IL-6 levels where not different. Of note, plasma LPS levels were similar in both genotypes (Table 2). Likewise, circulating TNFα levels were significantly lower in HFD-fed BM Fas-def mice (supplementary Table S1) and reduced in Fas-def ob/ob mice (4.5 ± 0.5 pg/ml in Fas-def ob/ob *vs*. 5.6 ± 0.9 pg/ml in WT ob/ob). Moreover, treatment of HFD-fed Fas^F/F^ and Fas^Δmye^ littermates with the TNFα blocking monoclonal antibody infliximab (Araujo *et al,* 2007), which recognizes both human as well as mouse TNFα epitopes (Qiu *et al,* 2011) abolished HFD-induced differences in glucose tolerance between both groups (Fig 6C) supporting the notion that circulating TNFα, induced by monocytic Fas, contributes to HFD-induced glucose intolerance.

### Human monocyte Fas expression correlates positively with serum LPS and TNFα

**Figure 7:**
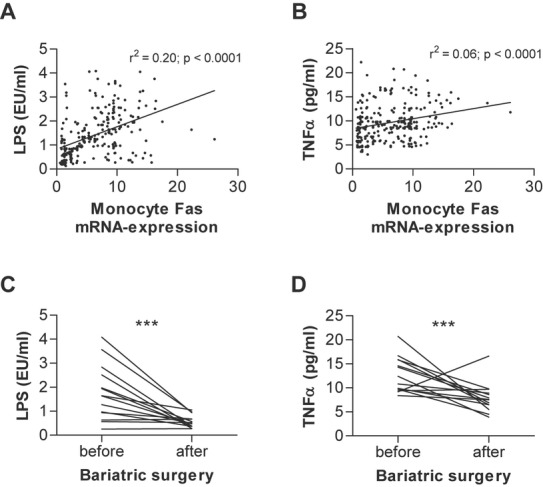
Human monocyte Fas expression positively correlates with serum LPS and TNFα levels. A,B  Monocyte Fas mRNA expression was determined and correlated to circulating LPS and TNFα levels. *n* = 246. C,D  Plasma LPS and TNFα levels were determined in obese patients before and 6 months after bariatric surgery (gastric sleeve resection; *n* = 14), ***p = 0.0007 (C) and ***p = 0.0009 (D) (Student's *t*-test).

In order to evaluate a potential involvement of TNFα in mediating the effect of monocyte-specific Fas on skeletal muscle insulin resistance, we determined circulating TNFα and LPS levels in the obese human cohort. Monocyte *Fas* mRNA positively correlated with circulating LPS (Fig 7A) and TNFα levels (Fig 7B). Multivariate linear regression models revealed that the correlations of monocytic *Fas* expression with LPS and TNFα serum concentrations are independent of age, gender and BMI (supplementary Table S2). In addition, weight loss in obese patients 6 months after bariatric surgery was associated with reduced circulating LPS and TNFα levels (Fig 7C and D) and BMI-adjusted changes in monocyte *Fas* expression significantly correlate with changes in circulating LPS (*r* = 0.14, *p* = 0.045) and TNFα (*r* = 0.15, *p* = 0.04). Jointly, these data support a role for monocyte-expressed Fas in the development of obesity-associated (muscle) insulin resistance in humans potentially via increased release of TNFα into circulation.

## Discussion

Unraveling inter-organ and inter-cell type specific crosstalk pathways underlying obesity-associated insulin resistance not only enhances our understanding of this complex disease process, but can yield new pharmaceutical interventions. The “inflammatory basis of obesity-associated morbidity” encompasses a particular challenge, since it involves activation of inflammatory cascades in multiple haematopoietic and non-haematopoietic cell types, and includes systemic and local markers of low-grade and chronic inflammatory activation that are all tightly inter-connected. The present study revealed a pathway in which myeloid cell-expressed Fas regulates systemic levels of TNFα. The latter in turn determines whole-body insulin sensitivity, particularly by modulating skeletal muscle insulin responsiveness. The major findings of this study supporting this proposition are: (i) myeloid cell-specific Fas disruption in mice results in lower circulating levels of TNFα, with improved whole-body insulin sensitivity that can be largely assigned to skeletal muscle; (ii) in obese human subjects, monocyte Fas expression correlates with circulating levels of TNFα and with glucose disposal rate. Demonstrating the protective effect of myeloid Fas ablation on skeletal muscle insulin sensitivity in the context of diet-induced obesity and leptin-deficiency as well as in response to LPS suggests the possibility that myeloid Fas is not an initiator of inflammation in obesity, but rather an ‘intermediate integrator’ that may respond to inflammatory cues like increased plasma LPS levels in the obese state. Thus, the myeloid cell—muscular crosstalk unraveled here is likely merely a segment of a more intricate pathway, in which myeloid Fas may constitute a critical node.

A specific example for the regulatory role of Fas is exemplified by the response to LPS. LPS was previously found to induce inflammatory cytokines in monocytes and macrophages, and interruption of Fas–FasL signalling suppressed nuclear factor-κB activation and cytokine expression induced by LPS in primary human and mouse macrophages (Ma *et al,* 2004). Complementarily, Fas activation induced a rapid release of pro-inflammatory cytokines in macrophages (Wang *et al,* 2010) and human monocytes (Park *et al,* 2003), which was dependent on toll-like receptor 4 (TLR4), a major receptor of LPS. We show here that LPS-induced up-regulation of Fas protein levels in myeloid cells significantly contributes to TNFα release. *In vivo*, high fat dieting (possibly via increased circulating LPS levels) increases myeloid Fas protein expression, and myeloid-specific Fas knockout reduces HFD- and LPS-induced circulating TNFα but not IL-6 levels. Hence, our data suggest that myeloid-cell expressed Fas plays an important role in the regulation of circulating TNFα levels. Moreover, our data provide evidence for a similar role of monocytic Fas in humans: monocyte Fas expression in obese people correlated with circulating LPS as well as TNFα concentrations and the drop in LPS levels in patients after bariatric surgery was paralleled by reduced monocytic Fas and circulating TNFα levels as well as improved insulin sensitivity. As previously reported, the composition of gut microbiota changes after bariatric surgery (Clement, 2011; Furet *et al,* 2010) and, thus, the observed improvement of glucose metabolism may at least in part be due to reduced endotoxemia and consequently reduced LPS levels (Manco *et al,* 2010). A role for Fas in metabolic diseases in human is further supported by a recent study revealing an association of Fas and FasL gene promoter polymorphisms with type 2 diabetes (Nolsoe *et al,* 2006).

TNFα has been among the first inflammatory mediators to be implicated in the induction of insulin resistance in obesity (Hotamisligil *et al,*^1993^). While TNFα infusion in healthy humans had no effect on endogenous glucose production, it induced skeletal muscle insulin resistance via inhibition of Akt substrate 160 phosphorylation (Plomgaard *et al,* 2005). Similarly, we found here that the decrease in circulating TNFα levels in mice with myeloid-specific deletion of Fas was associated with improved skeletal muscle but not liver insulin sensitivity. Moreover, neutralization of TNFα blunted HFD-induced differences in glucose tolerance between myeloid-specific Fas knockout and control mice, further supporting a causative role for TNFα in the observed phenotype. Furthermore, monocytic Fas expression in obese human individuals correlated positively with circulating TNFα concentration suggesting that the latter might be one of the downstream effectors of Fas up-regulation in myeloid cells in obesity-induced insulin resistance in human individuals. Of note, TNFα neutralization was reported to improve insulin resistance in diabetic patients with rheumatoid arthritis or Crohn's disease who were also receiving anti-TNFα treatment (infliximab) for their autoimmune disease (Gupta-Ganguli *et al,* 2011). Since we found no significant difference in immune cell infiltration and local cytokine expression in fat, liver and skeletal muscle between HFD-fed Fas^F/F^ and Fas^Δmye^ littermates, Fas expression in monocytes does not seem to impact on monocyte migration into peripheral tissue, supporting the idea that monocyte recruitment is mainly guided by recipient tissue rather than properties of circulating monocytes (Oh *et al,* 2012). Moreover, our findings would also suggest that myeloid cells rather than adipose tissue are the main source for circulating TNFα. Of note, the chosen period of high fat feeding (6 weeks) in our study is rather short. We cannot exclude that longer exposure to HFD may have led to a higher degree of adipose tissue inflammation and hepatic steatosis (Strissel *et al,* 2007), and, hence, myeloid-specific Fas deletion may have also influenced adipose tissue inflammation and/or hepatic insulin resistance.

The effect of LPS on cytokine expression is mainly mediated via TLR4, a pattern-recognition receptor known to regulate release of pro-inflammatory cytokines such as TNFα and IL-6 from monocytes/macrophages (Nguyen *et al,* 2007; Shi *et al,* 2006). As mentioned above, Fas was previously shown to modulate/enhance TLR4-mediated cytokine production (Ma *et al,* 2004). Hence, the beneficial effect of myeloid-specific Fas depletion on glucose tolerance may be merely due to reduced TLR4-signalling. However, myeloid/haematopoietic cell-specific TLR4 knockout mice as generated by adoptive BM transfer were not protected from HFD-induced skeletal muscle insulin resistance, whereas hepatic insulin sensitivity was significantly improved (supplementary Fig 19). Our data are in agreement with a previous study revealing blunted HFD-induced adipose and liver inflammation and ameliorated obesity-induced adipose and liver insulin resistance but no improvement in skeletal muscle insulin sensitivity in mice with haematopoietic cell-specific deletion of TLR4 (Saberi *et al,* 2009b). However, in contrast to this study (Saberi *et al,* 2009b), we found no difference in GIR between chimeras (supplementary Fig 19), which might be explained by different periods of HFD (6 weeks *vs*. 16 weeks). Of note, TNFα levels in HFD-fed haematopoietic cell-specific TLR4KO mice were comparable to wild-type chimeras (2.70 ± 0.29 pg/ml *vs*. 2.49 ± 0.3.5 pg/ml; *p* = 0.63).

In conclusion, our results reveal an important and unique role for myeloid cell expressed Fas in mediating obesity-induced insulin resistance in skeletal muscle. Pharmaceutical inhibition of Fas or its signalling pathway in myeloid cells may emerge as a promising new avenue in the treatment of insulin resistance and therefore type 2 diabetes.

## Materials and Methods

### Human samples

A total of 246 Caucasian men (*n* = 118) and women (*n* = 128) were selected among ∼1500 subjects recruited in the context of a study on insulin resistance at the Department of Medicine, University of Leipzig to represent a wide range of obesity, insulin sensitivity, and glucose tolerance. The age ranged from 19 to 80 years and BMI from 17.1 to 79.1 kg/m^2^. Subjects were subsequently divided into groups of NGT and type 2 diabetes (T2D) according to ADA-criteria (Anonymous, 2011). All study protocols have been approved by the ethics committee of the University of Leipzig. All participants gave written informed consent before taking part in the study.

#### Bariatric surgery study

Fourteen Caucasian obese volunteers (10 females, 4 males) participated in a prospective weight loss study before and 6 months after gastric sleeve resection. The baseline BMI was 54 ± 8 kg/m^2^ and the BMI 6 months after bariatric surgery was 36.3 ± 7.3 kg/m^2^. Individuals fulfilled the following inclusion criteria: (i) absence of any acute or chronic inflammatory disease as determined by a leukocyte count >7000 Gpt/L, C-reactive protein (CRP) >5.0 mg/dl or clinical signs of infection, (ii) undetectable antibodies against glutamic acid decarboxylase (GAD), (iii) no clinical evidence of either cardiovascular or peripheral artery disease, (iv) No thyroid dysfunction, (v) no alcohol or drug abuse, (vi) no pregnancy. BMI was calculated as weight divided by squared height. Hip circumference was measured over the buttocks; waist circumference was measured at the midpoint between the lower ribs and iliac crest.

#### Fas/FasL mRNA expression studies

Human monocytes were obtained from heparinized blood. Monocytes were separated using superparamagnetic polystyrene beads coated with a primary monoclonal antibody specific for the CD14 membrane antigen expressed on human monocytes (Invitrogen, Groningen, Germany) and resuspended in Hanks balanced solution containing (in mmol/L) NaCl 136, KCl 5.40, CaCl_2_ 1, KH_2_PO_4_ 0.44, Na_2_HPO_4_ 0.34, HEPES 10, pH 7.4. Total RNA was isolated from monocytes using the RNeasy mini kit including RNase-free DNase set (Qiagen, Hilden, Germany). Using the transcriptor first-strand cDNA synthesis kit (Roche Diagnostics, Mannheim, Germany), cDNA was synthesized from 2 μg of total RNA using oligo dT (12–18). Human *Fas* and *FasL* mRNA expression, determined by a premixed assay on demand (PE Biosystems, Darmstadt, Germany) was calculated relative to the mRNA expression of *HPRT* (PE Biosystems). The specificity of the PCR was verified by subjecting the amplification products to agarose gel electrophoresis.

#### Analysis of LPS, IL-6 and TNFα serum concentrations in human cohorts

All baseline blood samples were collected between 8 and 10 am after an overnight fast. Serum IL-6 and TNFα concentrations were measured by highly sensitive ELISAs for IL-6 (Quantikine IL-6, R&D Systems, Oxford, UK) and TNFα (Alpco Diagnostics, Salem, NH, USA). Serum LPS concentration was measured using a LAL Chromogenic Endpoint Assay (Hycult Biotech Inc., PB Uden, The Netherlands).

### Animals

Total body Fas-deficient (Fas-def) mice on C57BL/6J inbred strain background (B6.MRLFas^lpr^) were obtained from The Jackson Laboratory. C57BL/6J mice with exon IX of Fas flanked with LoxP sites were produced as described (Stranges *et al,* 2007) and crossed to LysozymeM (LysM)-Cre mice (B6,129-Lys<tm1(Cre)Ifo>) to generate myeloid-specific deletions (Fas^Δmye^). All mice were housed in a specific pathogen-free environment on a 12-h light-dark cycle and fed *ad libitum* with regular chow diet (Provimi Kliba) or HFD (58 kcal% fat w/sucrose Surwit Diet, D12331, Research Diets). All protocols conformed to the Swiss animal protection laws and were approved by the Cantonal Veterinary Office in Zurich, Switzerland.

#### Genotyping of animals

All mice were genotyped by PCR with primers amplifying the Cre transgene (generating 350 bp wild-type and 700 bp Cre allele products) and Fas (generating 319 bp wild-type and 399 bp ‘floxed’ allele products).

#### Bone marrow transfer

BM chimeras were generated by either transplanting 2 × 10^6^ B6.MRLFas^lpr/J^ CD45.2 (FAS-KO), Tlr4^tm1Aki^ (CD45.2) or C57BL/6J CD45.2 (control) total BM cells into lethally irradiated (2 × 450 cGy) 6-week-old B6.SJL CD45.1 or 6-week-old B6.V-Lep^ob^/OlaHsd recipient mice. Recipient mice were maintained and bled at week 6 to check the chimerism in peripheral blood and a > 90% donor chimerism was confirmed in peripheral blood of all recipient animals.

#### Metabolic cage analysis

Locomotion, food and water intake, O_2_ consumption and CO_2_ production were determined for single housed mice during a 24-h period in a metabolic and behavioural monitoring system (PhenoMaster, TSE Systems, Bad Homburg, Germany).

#### Intra-peritoneal glucose and insulin tolerance tests

For the intraperitoneal glucose tolerance test (ipGTT) mice were either fasted for 5 h (ob/ob mice) or overnight (C57BL/6J), for the intraperitoneal insulin tolerance tests (ipITT) mice were fasted for 3 h. Either glucose (0.25 g/kg body weight for ob/ob mice; 2 g/kg body weight for C57BL/6J) or human recombinant insulin (0.75 U/kg body weight) were injected intraperitoneally (Konrad *et al,* 2007).

#### Glucose clamp studies

Glucose clamp studies were performed as described (Rytka *et al,* 2011). Clamps were performed in freely moving mice. Glucose infusion rate was calculated once glucose infusion reached a more or less constant rate with blood glucose levels at 5 mmol/L (80–90 min after the start of insulin infusion). Thereafter, blood glucose was kept constant at 5 mmol/L for 20 min and glucose infusion rate was calculated. The glucose disposal rate was calculated by dividing the rate of [3-^3^H]glucose infusion by the plasma [3-^3^H]glucose specific activity (Fisher & Kahn, 2003; Kim *et al,* 2000). Endogenous glucose production during the clamp was calculated by subtracting the glucose infusion rate from the glucose disposal rate (Fisher & Kahn, 2003; Kim *et al,* 2000). Insulin-stimulated glucose disposal rate was calculated by subtracting basal endogenous glucose production (equal to basal glucose disposal rate) from glucose disposal rate during the clamp (Saberi *et al,* 2009a).

#### Glucose incorporation into isolated soleus muscle

Intact soleus muscle was isolated and thereafter incubated with or without 2 mU of insulin per ml. 2-Deoxyglucose uptake was then measured for 20 min as described previously (Rudich *et al,* 2003).

#### Determination of plasma insulin, adipokines, free fatty acid and triglyceride levels

Plasma insulin and FFA levels were determined as described (Konrad *et al,* 2007). Plasma leptin and cytokine levels were determined with mouse procarta cytokine assay kit (Affymetrix). Plasma adiponectin levels were measured with an ELISA kit (Axxora). TG concentrations were determined using a colorimetric assay (Sigma).

#### TNFα neutralization

Mice were treated with infliximab (Remicade®, MSD) for 8 days by daily injections (i.p.) of 50 μg infliximab dissolved in 100 μl saline.

#### Stromal vascular fraction (SVF), muscle and liver cell isolation for flow cytometry

After digestion of adipose tissue (Rudich *et al,* 2003), isolated cells were centrifuged at 4°C (500 g) for 5 min. SVF (pellet) was kept on ice in KRP 0.1% BSA. Centrifugation step was repeated with infra- and supernatant for three times and obtained SVFs were pooled and dissolved in PBS 0.1% BSA. For isolation of muscle cells, skeletal muscle was minced in PBS and kept on ice. After centrifugation, 5 ml collagenase solution (0.5 ml 10 ×  HBSS, 4.5 ml H_2_O, 75 mg BSA, 10 mg collagenase II (Life Technologies)) was added to the pellet and shaken in a water bath at 37°C for 20 min. Thereafter, cells were centrifuged (80 g) and supernatant was poured into 15 ml ice-cold media (DMEM low glucose, 10% FBS). Digestion of the pellet was repeated twice. Subsequently, cells were filtered through a cell restrainer (100 μm), centrifuged (350 g), resuspended in erythrocyte lysis buffer and filled up with ice-cold PBS to 25 ml. After filtering through a 40 μm cell restrainer, cell pellet was dissolved in PBS 0.1% BSA. For liver cell isolation, livers were mashed between glass slides and cells were suspended in FACS buffer. Cell suspension was layered on Ficoll and centrifuged (400 g) for 30 min at 18°C. Cells on top of Ficoll were collected and washed in FACS buffer.

#### Flow cytometric analysis

For flow cytometric analysis of isolated SVF, muscle and liver cells, cells were blocked with Fc blocker (anti CD16/32 BD pharmingen) on ice for 15 min. Thereafter, cells were stained on ice for 30 min with the following antibodies: CD206-Alexa Fluor 488, CD11c-PE Cy7 (both from Biolegend), CD45.2-PE, CD8-PE-Cy5, CD4-APC (all from eBioscience), F4/80-Fluor 780 (Alexa). Cells were subsequently washed for three times and resuspended in Hoechst buffer. For flow cytometry analysis of circulating blood cells, mice were bled and red blood cells were lysed using ACK lysis buffer (150 mM NH_4_Cl, 10 mM KHCO_3_, 0.1 mM EDTA). Cell debris was removed by filtering the cell suspension using a 70 μm cell strainer (BD). Peripheral leukocytes were stained using fluorochrome conjugated antibodies as follows: Pacific Blue-conjugated anti-CD45.1 (clone A20; Biolegend), FITC-conjugated anti-GR-1 (clone RB6-8C5; ebioscience.com), PE-conjugated anti-FAS (CD95; clone Jo2; BD-Pharmingen), PE-Cy5-conjugated anti-CD45.2 (clone 104; ebioscience), APC-conjugated anti-CD11c (clone M1/70; ebioscience), eFluor 780 conjugated anti-CD11b (clone M1/70; ebioscience) and Hoechst 33342 2 μg/ml to exclude dead cells. Immunophenotypical analysis was performed on a BD FACS Canto II Flow Cytometer.

#### Histology

Fat tissues were fixed in 4% buffered formalin and embedded in paraffin. Sections were cut and stained with haematoxylin and eosin. For each fat pad of a mouse, at least 100 adipocytes were analysed (ImageJ software; National Institutes of Health, Bethesda, MD).

#### RNA extraction and quantitative reverse transcription-PCR (RT-PCR)

Total RNA was extracted and reverse transcribed as described (Wueest *et al,* 2010b). The following primers were used: TNF-α Mm00443258_m1, IL-6 Mm00446190_m1, IL-1β Mm0043422/8_m1, cd11b Mm00434455_m1, cd11c Mm00498698_m1, cd68 Mm03047343_m1, F4/80 Mm00802529_m1, MCP-1 Mm00441242_m1, IL-1β Mm0043422/8_m1, IL-10 Mm00439614_m1, FAS Mm00662319_m1, ACC-1 Mm01304289_m1, CPT-1 Mm00550438_m1, AOX Mm00443579_m1, PGC-1α Mm01208835_m1, FasL Mm00438864_m1 (Applied Biosystems).

#### Western blotting

Cells or tissues were lysed and Western blots were performed as previously described (Wueest *et al,* 2010b). The following primary antibodies were used: anti-Fas (clone 7C10), anti-phospho-AS160 (Thr642) and anti-actin (Millipore), anti-phospho-Akt (Thr308; Cell Signaling).

### Cell culture experiments

RAW 264.7 cells were grown in RPMI 1640, 1% penicillin/streptomycin (p/s), 1% glutamine and 10% FCS. At 30–50% confluence, cells were treated with siRNA (target sequences: 5′ GAACCATTATGCTGATAAA 3′, 5′ AAAGCCGAATGTCGCAGAA 3′; scrambled sequences: 5′ GACACTAAACGTGAATTAT 3′, 5′ GCAACAGGCGTCAAGAAAT 3′) in a transfection mixture containing Lipofectamin 2000 (Invitrogen) in RPMI (without FCS, without p/s) according to the manufacturer's instructions. L6 myotubes differentiation and glucose uptake were performed as described (Niu *et al,* 2003). For TNFα neutralization, L6 myotubes were incubated with nTNFα or IgG control antibody (R&D Systems). Glucose uptake in mature 3T3-L1 adipocytes was performed as described (Wueest *et al,* 2010a). HepG2 cells were cultivated in DMEM supplemented with 10% FCS.

### Data analysis

#### Animal data

Data are presented as means ± SEM and were analysed by two-tailed Student's *t*-test, one sample *t*-test or one-way ANOVA with a Tukey correction for multiple group comparisons. *p* Values <0.05 were considered significant.

#### Human cohort data

Data are shown as means ± SEM unless stated otherwise. Before statistical analysis, non-normally distributed parameters were logarithmically transformed to approximate a normal distribution. The following statistical tests were used: paired Student's *t*-test, Chi-square test, and Pearson's simple correlation. Prediction models for monocyte *Fas* mRNA expression based on age, gender, anthropometric and clinical parameters were calculated by multivariate linear regression analysis. *p* Values <0.05 were considered to be statistically significant. Statistical analysis was performed using SPSS version 12.0 (Chicago, IL).
