# Supplementary material for: Fas (CD95) expression in myeloid cells promotes obesity-induced muscle insulin resistance
Source: EMBO Mol Med. 2013 Nov 6;6(1):43–56. doi: 10.1002/emmm.201302962 (PMC3936487; doi:10.1002/emmm.201302962)
Supplement: Supplementary file 1 [file emmm0006-0043-sd1.pdf]

EMBO
Molecular Medicine

Fas (CD95) expression in myeloid cells promotes obesity-induced muscle insulin resistance

Stephan Wueest, Rouven Mueller, Matthias Blüher, Flurin Item, Annie S.H. Chin, Michael S. F. Wiedemann, Hitoshi Takizawa, Larisa Kovtonyuk, Alexander V. Chervonsky, Eugen J. Schoenle, Markus G. Manz and Daniel Konrad

Corresponding author: Daniel Konrad, University Children's Hospital, Zurich

Review timeline:

Submission date:	27 April 2013
Editorial Decision:	26 June 2013
Revision received:	24 September 2013
Editorial Decision:	30 September 2013
Revision received:	30 September 2013
Accepted:	01 October 2013

Transaction Report:

(Note: With the exception of the correction of typographical or spelling errors that could be a source of ambiguity, letters and reports are not edited. The original formatting of letters and referee reports may not be reflected in this compilation.)

Editor: Roberto Buccione

1st Editorial Decision 26 June 2013

Thank you for the submission of your manuscript to EMBO Molecular Medicine.

As you know, in this case we experienced some difficulties in obtaining a third and last evaluation from the assigned Reviewer. Since we could not justify a further delay, I have made a decision based on the two available and somewhat conflicting evaluations, after having obtained external advice from an Editorial Board member.

You will see that while Reviewer 1 is generally supportive of your work and underlines its considerable potential interest, Reviewer 3 is more critical and raises a number of specific concerns that question the validity of your work thus preventing us from considering publication at this time.

Reviewer 1 suggests a number of points to improve the overall solidity and impact of the manuscript. Firstly s/he would like to know if CD95 is also up-regulated in other tissues in the obese state. Reviewer 1 also asks whether there is a change in circulating or myeloid CD95L between Fas mice and controls and wonders whether CD95L can be assessed in patient plasma.

Reviewer 3 expresses a number of important concerns that require your action. S/he would like you to perform a longer (i.e. 20 week) high fat study to strengthen your conclusion that myeloid Fas affects the muscle, but not other organs. I understand that this would be time-consuming and might not add much to the overall message; we would thus suggest that you clarified that a model of short duration of a dietary intervention was employed, carefully discussed the possible implications in a longer setting and better synthesised the results from the models employed. Reviewer 3 also suggests that flow cytometry is required to assess cell infiltration. We agree that this is warranted if conclusions are being made on the infiltrating cell types. If, however, the point being made were

about adipose tissue inflammation, expression profiles of markers combined with immunohistochemistry would be needed. Clear clarification, and subsequent appropriate actions, are required to resolve this specific point. The remaining important issues raised by Reviewer 3 should be fully addressed.

While publication of the paper cannot be considered at this stage, we would be pleased to consider a suitably revised submission, provided, however, that the Reviewers' concerns are fully addressed (according to the provisions specified above) with additional experimental data where appropriate.

Please note that it is EMBO Molecular Medicine policy to allow a single round of revision only and that, therefore, acceptance or rejection of the manuscript will depend on the completeness of your responses included in the next, final version of the manuscript.

As you know, EMBO Molecular Medicine has a "scooping protection" policy, whereby similar findings that are published by others during review or revision are not a criterion for rejection. However, I do ask you to get in touch with us after three months if you have not completed your revision, to update us on the status. Please also contact us as soon as possible if similar work is published elsewhere.

I look forward to receiving your revised manuscript.

***** Reviewer's comments *****

Referee #1 (Remarks):

The CD95/CD95L system plays an important role in regulating tissue homeostasis, primarily by inducing cell death. Only recently it has become more and more clear that CD95 exerts several non-apoptotic functions.

Wueest and coauthors studied the role of CD95 in obesity.
Obesity is considered a state of chronic low grade inflammation and is, for example, characterized by the infiltration of macrophages into adipose tissue, which subsequently gives rise to insulin resistance and other obesity-related disorders. Since the CD95 system is implicated in the regulation of immune responses the authors were interested in understanding the role of CD95 in myeloid cells in the context of obesity.

They found CD95 upregulated in monocytes from obese glucose tolerant subjects compared to obese diabetic subjects. CD95 levels correlated with measures of systemic and muscular insulin resistance. Encouraged by this they generated transgenic mice with a myeloid-specific knockout of CD95. In line with human data, these animals were protected from HFD-induced glucose intolerance with clamp assays showing that this was mainly due to improved muscle insulin sensitivity. Findings were further corroborated in a second model system, where wt or genetically obese mice received a bone marrow transplantation from CD95-knockout mice yielding animals with absence of CD95 in all hematopoietic cells. Also these animals were protected from obesity-induced muscle insulin resistance. Finally, the authors provide experimental evidence that TNFalpha is the molecule linking the function of myeloid CD95 and muscle insulin resistance.

This is a very good manuscript. The experimental outline is straightforward. The manuscript is clear, structured and well written. The model systems are well chosen: findings from sophisticated animal models are combined with human data from very well characterized patient cohorts. The topic of this work is highly relevant for both the obesity and the death receptor field.

Major points:

1. Is there a difference in CD95 in monocytes between lean and obese subjects?

2. CD95 is upregulated in obese compared to lean WAT. This study shows that CD95 is upregulated in diabetic vs non-diabetic monocytes. Is CD95 upregulation a general feature of inflammation or stress?

The CD95 system is upregulated in affected tissues in several autoimmune diseases, e.g. T1DM and Hashimoto thyroiditis. It would be interesting to know if CD95 is upregulated also in other tissues in the obese state, such as muscle, liver, other immune cells ...

3. The authors clearly show that myeloid CD95 is an important regulator of muscle insulin sensitivity. CD95 is activated by CD95L. What do the authors think happens in the context of obesity? CD95L is usually membrane bound, but can also be secreted to the circulation.
In that sense: Is there a change in circulating or myeloid-expressed CD95L between Fas mye and control mice? What is monocyte CD95L expression in patient samples? Would it be possible to measure CD95L in patient plasma?

4. Are SNPs or mutations in CD95 associated with a diabetic phenotype?

5. Which subtypes of myeloid cells are affected by the knockout? Monocytes? Macrophages? Dendritic cells? Myeloid-derived suppressor cells?

Referee #3 (Comments on Novelty/Model System):

The are two major problems in the model used:

The authors performed a very brief model of diet-induced obesity (6 weeks). This is the earliest time point where e.g. inflammation in the adipose tissue starts playing a role. Thus, it is important to perform a longer study (e.g. 16-20 weeks) of diet-induced obesity analyzing the liver, adipose tissue and the muscle in order to really back their conclusion that only the muscle but not other organs are affected by myeloid Fas.

In addition, the authors conclude that myeloid cell Fas does not regulate immune cell infiltration or inflammation in the liver, adipose tissue or muscle by qPCR analysis. This method is inappropriate to assess cell infiltration. Flow cytometry is needed for this purpose (after 6 and especially 16 weeks of feeding), with a thorough analysis of at least T cells (CD4, CD8) and macrophages (including M1 and M2 macrophages).

Referee #3 (Remarks):

In this manuscript the authors addressed the role of myeloid cell Fas in insulin resistance. In obese individuals, monocyte Fas expression correlated with insulin resistance. Mice with myeloid Fas deficiency either by using conditional mice or bone marrow chimeras displayed reduced glucose intolerance and insulin resistance in diet-induced or genetically-induced obesity, or in LPS-treated mice. The authors suggest that myeloid Fas only affects insulin resistance of the skeletal muscle but not of the liver or the adipose tissue and that this effect is mediated by TNF.

A problem of the manuscript is that this major conclusion of the paper is not supported well by the data. While the role of myeloid Fas in insulin resistance is a potentially very interesting finding, it is not clear how myeloid Fas is linked to insulin resistance specifically of the skeletal muscle and not of the liver or the adipose tissue. That TNF affects insulin resistance in many tissues including the adipose tissue is well established. Since the authors suggest that there is no difference in local tissue inflammation in the muscle by Fas deficiency but they find only circulating TNF levels to be regulated by Fas, then one would expect circulating TNF to mediate insulin resistance not only in the muscle but in other insulin target organs as well. How can an increase in circulating TNF levels (which will affect several organs) confer specificity only to the skeletal muscle but not the other organs, e.g. the adipose tissue? This is very difficult to understand. A possible explanation is that the authors performed a very brief model of diet-induced obesity (6 weeks). This is the earliest time point where e.g. inflammation in the adipose tissue starts playing a role. Thus, it is important to perform a longer study (e.g. 16-20 weeks) of diet-induced obesity analyzing the liver, adipose tissue and the muscle in order to really back their conclusion that only the muscle but not other organs are affected by myeloid Fas.

Along the same line, is the regulation of TNF levels in the supernatant of macrophages (figures 4) responsible for the effect of myeloid Fas on myotubes (figure 4F)? The authors should immuno-deplete TNF from their supernatant to address this question.

Why do the authors use RAW cells in figure 4? As they have both complete and myeloid-specific Fas ko mice, they should isolate thioglycollate-elicited macrophages or bone marrow derived macrophages from wt and ko mice and perform these assays.

In addition, the authors conclude that myeloid cell Fas does not regulate immune cell infiltration or inflammation in the liver, adipose tissue or muscle by qPCR analysis (suppl. fig. 9 and 10). This method is inappropriate to assess cell Infiltration. Flow cytometry is needed for this purpose (after 6 and especially 16 weeks of feeding), with a thorough analysis of at least T cells (CD4, CD8) and macrophages (including M1 and M2 macrophages).

In fact, the question whether myeloid Fas regulates M1 vs M2 polarization of macrophages needs to be addressed. For this purpose, thioglycollate-elicited wt and ko macrophages should be treated with LPS+/- IFN or IL-4 and the expression of TNF, IL-6, iNOS, IL-10, YM1 etc. should be studied.

Moreover, the authors conclude in the discussion that their data suggest "...the possibility that myeloid Fas is not an initiator of inflammation in obesity, but rather an "intermediate integrator" that may respond to inflammatory cues like those generated by the gut". This conclusion is speculative. There is no gut or microbiome analysis in the paper in wt and myeloid-specific Fas ko mice under normal and high fat diet conditions. Their conclusion is based on the LPS data. However, that LPS regulates Fas is well established and these data do not really add much novelty to the paper.

1st Revision - authors' response 24 September 2013

Response to Editor

We thank the Editor for his insightful and helpful guidance how to revise our manuscript.

Reviewer 1 suggests a number of points to improve the overall solidity and impact of the manuscript. Firstly s/he would like to know if CD95 is also up-regulated in other tissues in the obese state. Reviewer 1 also asks whether there is a change in circulating or myeloid CD95L between Fas mice and controls and wonders whether CD95L can be assessed in patient plasma.

As outlined in the response to Reviewer #1, CD95 is not up regulated in skeletal muscle, liver, neutrophils as well as B- and T- lymphocytes of obese compared to lean mice. Moreover, there was no significant difference in CD95L expression in circulating myeloid cells between myeloid-specific Fas knockout and control mice.
As for CD95L, its expression was higher in monocytes of obese persons with type 2 diabetes compared to obese, normal glucose tolerant subjects. In patient plasma, a recent publication (Choi JW and Kim SK. Ann Clin Lab Sci 2005: 35(3): 290-6) showed that sCD95L is not increased in serum of obese compared to lean human subjects.

Reviewer 3 expresses a number of important concerns that require your action. S/he would like you to perform a longer (i.e. 20 week) high fat study to strengthen your conclusion that myeloid Fas affects the muscle, but not other organs. I understand that this would be time-consuming and might not add much to the overall message; we would thus suggest that you clarified that a model of short duration of a dietary intervention was employed, carefully discussed the possible implications in a longer setting and better synthesised the results from the models employed. Reviewer 3 also suggests that flow cytometry is required to assess cell infiltration. We agree that this is warranted if conclusions are being made on the infiltrating cell types. If, however, the point being made were about adipose tissue inflammation, expression profiles of markers combined with immunohistochemistry would be needed. Clear clarification, and subsequent appropriate actions, are required to resolve this specific point. The remaining important issues raised by Reviewer 3 should be fully addressed.

In the revised manuscript, we clarified that a model of short duration of high fat diet was employed and we discuss that longer periods of high fat feeding may differently affect the observed phenotype. To analyse immune cell infiltration, we performed flow cytometric analysis of T cells and macrophages in skeletal muscle, white adipose tissue and liver of FasF/F and FasΔmye mice fed a high fat diet for 6 weeks. We found neither a difference in immune cell infiltration nor in macrophage polarization between control and myeloid-specific Fas knockout mice in any tissue analysed. Moreover, we addressed the remaining important issues raised by Reviewer 3 as outlined below.

Point by Point Response to the Reviewers' Comments

Responses to Reviewer #1

We thank the reviewer for his/her insightful comments and we are happy to learn that he/she found the manuscript clear and well written and the topic of the work highly relevant for the obesity and the death receptor field.

Major points:

1. Is there a difference in CD95 in monocytes between lean and obese subjects?

To unravel whether obesity has an impact on myeloid Fas expression, mRNA levels were determined in circulating monocytes of lean and obese subjects (BMI: 21.4±0.5 kg/m2 in lean vs. 45.9±1.1 kg/m2 in obese subjects, p<0.0001). We found that Fas expression was significantly increased in obese compared to lean human subjects. Such finding was added to the revised manuscript (Fig. 1A).

2. CD95 is upregulated in obese compared to lean WAT. This study shows that CD95 is up regulated in diabetic vs non-diabetic monocytes. Is CD95 up regulation a general feature of inflammation or stress?
The CD95 system is up regulated in affected tissues in several autoimmune diseases, e.g. T1DM and Hashimoto thyroiditis. It would be interesting to know if CD95 is up regulated also in other tissues in the obese state, such as muscle, liver, other immune cells ...

While CD95 was up regulated in monocytes of diabetic compared to non-diabetic subjects (Fig.1B of revised manuscript), we found no difference in CD95 mRNA expression in skeletal muscle and livers between diabetic and non-diabetic human beings (see Figures below).
NGTT2D0.00.51.01.52.0Muscle FasmRNA-expressionNGTT2D0246810Liver FasmRNA-expression

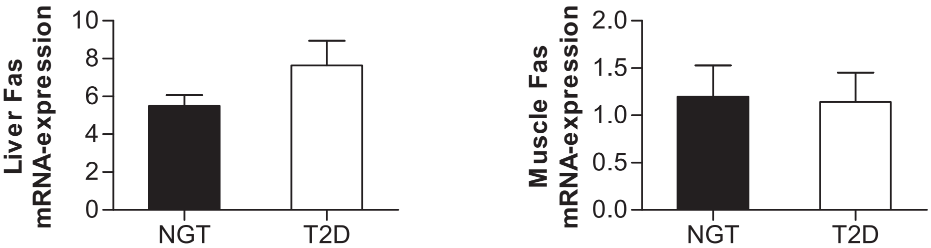

Liver and muscle Fas mRNA expression
Fas mRNA expression was measured and normalized to HPRT. NGT: normal glucose tolerance ; T2D: type 2 diabetes. n=16-40. Error bars represent SEM.

Hence, this data suggests that CD95 is not generally up regulated in any tissue of type 2 diabetic patients.

In mice, Fas protein could not be detected in skeletal muscle of obese FasF/F and FasΔmye mice (see Western blot below). Moreover, Fas mRNA was not increased upon HFD in skeletal muscle of

C57BL/6J mice (Figure 3G of submitted manuscript).

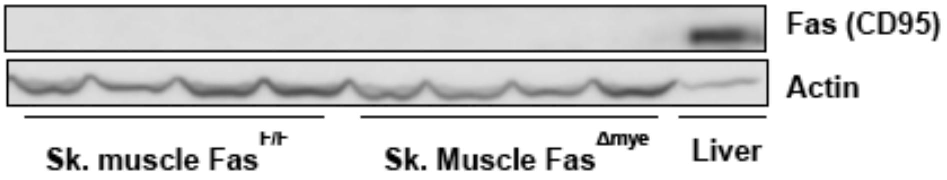

In livers of C57BL/6J mice, high fat feeding for 6 weeks had no effect on Fas protein levels (see Figure below), which is in accordance with previous findings showing similar Fas protein levels in livers of obese compared to lean mice (Zou C et al. Nat Med. 2007 Sep; 13(9):1078-85).

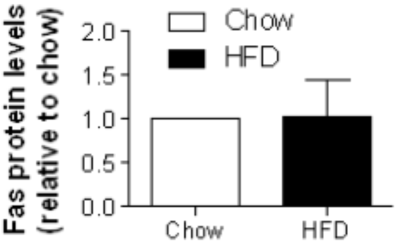

Liver Fas protein levels in chow and HFD C57BL/6J mice
Total liver lysates were prepared from wild-type C57Bl6/J mice fed either a chow or a high fat diet for six weeks. Lysates were resolved by LDS-PAGE and immunoblotted with anti-Fas antibody. Results are the mean ± SEM of 3 mice per group.

Moreover, HFD did not increase Fas protein levels neither in B- and T-cells nor in neutrophils. We added this finding to the revised manuscript (Supplemental Fig. 5). Hence, this data suggests that obesity does not lead to a general up regulation of Fas (CD95) in mice.

3. The authors clearly show that myeloid CD95 is an important regulator of muscle insulin sensitivity. CD95 is activated by CD95L. What do the authors think happens in the context of obesity? CD95L is usually membrane bound, but can also be secreted to the circulation.
In that sense: Is there a change in circulating or myeloid-expressed CD95L between Fas mye and control mice? What is monocyte CD95L expression in patient samples? Would it be possible to measure CD95L in patient plasma?

FasL expression was similar in HFD-fed control and myeloid-specific Fas knockout mice. In contrast, TNFα expression was significantly lower in circulating immune cells of FasDmye mice, supporting findings in LPS-injected mice (Fig. 5B of submitted manuscript). We added data of myeloid FasL and TNFα expression to the revised manuscript (Fig. 6A and Supplemental Fig. 8). As suggested, monocyte CD95L expression was measured in patient samples. FasL expression was higher in monocytes of obese persons with type 2 diabetes compared to obese, normal glucose tolerant subjects. Such finding was added to the revised manuscript (Supplemental Fig. 1). As for CD95L in patient plasma, a recent publication (Choi JW and Kim SK. Ann Clin Lab Sci 2005: 35(3): 290-6) showed that sCD95L is not increased in serum of obese compared to lean human subjects.

4. Are SNPs or mutations in CD95 associated with a diabetic phenotype?

Studies in humans revealed that promoter polymorphisms of the Fas and FasL gene are associated with type 2 diabetes (Nolsoe RL et al. Genes Immun. 2006; 7(4): 316-321). We added this point to the discussion section of the revised manuscript.

5. Which subtypes of myeloid cells are affected by the knockout? Monocytes? Macrophages? Dendritic cells? Myeloid-derived suppressor cells?

LysM-Cre mediated loxP site recombination efficiency was reported to be 83-98% in mature macrophages and near 100% in granulocytes. Moreover, partial deletion (16-31%) was detected in

dendritic cells (Clausen BE et al. Transgenic Research 1999; 8: 265-277). This is in accordance with data showing that LysM reporter mice show some eGFP expression in DC subsets and high expression in monocytes (Liu K et al. Science 2009; 324: 392-7). We adapted the statement regarding LysM-Cre expression in the revised manuscript.

Responses to Reviewer #3

We thank the reviewer for his/her constructive comments and raised questions.

This is the earliest time point where e.g. inflammation in the adipose tissue starts playing a role. Thus, it is important to perform a longer study (e.g. 16-20 weeks) of diet-induced obesity analyzing the liver, adipose tissue and the muscle in order to really back their conclusion that only the muscle but not other organs are affected by myeloid Fas.

We thank the reviewer for this well taken point. However, as suggested by the editor, we did not perform the time-consuming experiment with a longer exposure to HFD, but rather carefully discuss the possible implications of a longer HFD period in the discussion section of the revised manuscript.

Along the same line, is the regulation of TNF levels in the supernatant of macrophages (figures 4) responsible for the effect of myeloid Fas on myotubes (Figure 4F)? The authors should immuno-deplete TNF from their supernatant to address this question.

The inhibitory effect of conditioned medium from LPS-treated RAW cells on insulin-stimulated glucose uptake in L6 myotubes was blunted upon TNFα neutralization indicating that the effect of such conditioned media on insulin resistance is TNFα dependent. We added such finding to the revised manuscript (Figure 5G).

Why do the authors use RAW cells in figure 4? As they have both complete and myeloid-specific Fas ko mice, they should isolate thioglycollate-elicited macrophages or bone marrow derived macrophages from wt and ko mice and perform these assays.

We thank the Reviewer for this suggestion, which surely would be a good alternative to the experiments performed with RAW cells. However, in order to reduce the number of animals (according the 3R ethical framework (replace, reduce, refine)), we aimed to perform additional experiments supporting a role of myeloid Fas expression on skeletal muscle insulin sensitivity in cell lines *in vitro*. Hence, we decided to use L6 myotubes (instead of primary cultures of skeletal muscle cells) and RAW cells, a cell line reported to be a good model to study inflammation-related mechanism (Maurya MR et al. J Lipid Res 2013; 54(9): 2525-42). As we were able to almost completely reduce Fas protein levels in RAW cells by siRNA (Fig. 5D of submitted manuscript), we thought to have a good *in vitro* tool for such experiments.

In addition, the authors conclude that myeloid cell Fas does not regulate immune cell infiltration or inflammation in the liver, adipose tissue or muscle by qPCR analysis (suppl. fig. 9 and 10). This method is inappropriate to assess cell Infiltration. Flow cytometry is needed for this purpose (after 6 and especially 16 weeks of feeding), with a thorough analysis of at least T cells (CD4, CD8) and macrophages (including M1 and M2 macrophages).

We thank the Reviewer for this important point. As suggested, we performed flow cytometry analysis of T cells and macrophages in skeletal muscle, white adipose tissue and liver of FasF/F and FasΔmye mice fed a high fat diet for 6 weeks. As presented in the revised manuscript (Supplemental Fig. 12E-G, Supplemental Fig. 13B), we did not find a difference neither in immune cell infiltration nor in macrophage polarization between control and myeloid-specific Fas knockout mice in any tissue analysed.

In fact, the question whether myeloid Fas regulates M1 vs M2 polarization of macrophages needs to be addressed. For this purpose, thioglycollate-elicited wt and ko macrophages should be treated with LPS+/- IFN or IL-4 and the expression of TNF, IL-6, iNOS, IL-10, YM1 etc. should be studied.

We analysed mRNA expression of TNFα, IL-6, IL-10 and Arginase 1 in thioglycollate-elicited, M1 and M2 polarized macrophages from FasF/F and FasΔmye mice. As shown below, myeloid Fas knockout decreased M1 macrophage polarization, whereas it has a minor effect on M2 polarization. This data is consistent with reduced LPS stimulated TNFα release/expression in myeloid cells (Figures 5B/C/E of submitted manuscript).

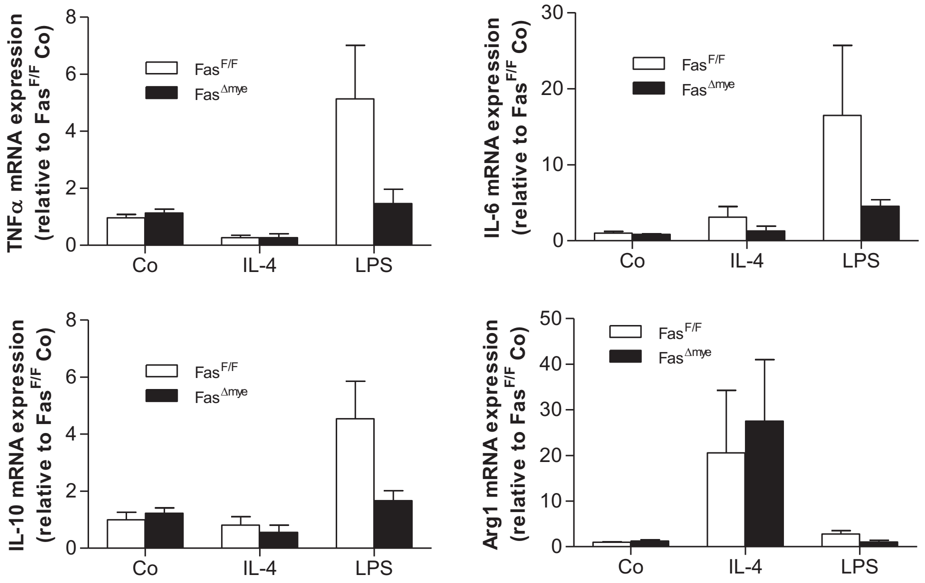

mRNA expression of thioglycollate-elicited M1 and M2 polarized macrophages
Thioglycollate-elicited macrophages were eluted from the peritoneum of FasF/F (white bars) and FasDmye (black bars) mice. Subsequently, macrophages were stimulated *ex-vivo* with 100 ng/ml LPS or 20 ng/ml IL-4 for 24 hours. Thereafter, mRNA expression was determined. n=3.

Moreover, the authors conclude in the discussion that their data suggest "...the possibility that myeloid Fas is not an initiator of inflammation in obesity, but rather an "intermediate integrator" that may respond to inflammatory cues like those generated by the gut". This conclusion is speculative. There is no gut or microbiome analysis in the paper in wt and myeloid-specific Fas ko mice under normal and high fat diet conditions. Their conclusion is based on the LPS data. However, that LPS regulates Fas is well established and these data do not really add much novelty to the paper.

We agree with the Reviewer that our conclusion is not based on gut analysis but rather on LPS. We therefore adapted the discussion in the revised manuscript to “the possibility that myeloid Fas is not an initiator of inflammation in obesity, but rather an "intermediate integrator" that may respond to inflammatory cues like increased plasma LPS levels in the obese state.

2nd Editorial Decision 30 September 2013

Thank you for the submission of your revised manuscript to EMBO Molecular Medicine. As one of the Reviewers (#3) whom we had asked to re-evaluate your revised manuscript was not available, we asked Reviewer 1 to do so in his/her lieu.

I am pleased to inform you that the Reviewer is fully supportive, as you will see below, and that we will be able to accept your manuscript pending the following final amendments:

1) As per our Author Guidelines, the description of all reported data that includes statistical testing must state the name of the statistical test used to generate error bars and P values, the number (n) of independent experiments underlying each data point (not replicate measures of one sample), and the actual P value for each test (not merely 'significant' or 'P < 0.05').

2) Please change all supplementary information figures from their current landscape orientation to a portrait (i.e. vertical) format.

3) Please submit a manuscript file without the yellow highlighting (no longer needed).

I look forward to receiving the next, final version of your manuscript as soon as possible and in any case within two weeks. Obviously, the sooner we receive it, the sooner we will be able to proceed with formal acceptance!

***** Reviewer's comments *****

Reviewer #1 (Remarks):

My concerns have been addressed in sufficient detail. I have no further comments and think that the manuscript is now suitable for publication.

Reviewer #1 (Remarks on Reviewer 3's concerns):

I carefully went through all comments raised by Reviewer #3.

This is my final opinion on the manuscript by Wueest et al:
The authors generated mice with myeloid-specific knockout of CD95 and demonstrate that these animals are protected from glucose intolerance with specific effects on muscle. They used a bone marrow transfer model to further corroborate their findings. They performed quite a lot of in vitro work to unravel the underlying molecular mechanism of their findings. On top of all that, authors studied CD95 and CD95L expression in a unique patient cohort of >100 healthy and >100 diabetic patients.

I think that they fully made their point and that all concerns by Reviewer #3 were addressed appropriately.

Taken together I think that the work by Wueest et al. is acceptable for publication.
