# Supplementary material for: Fas (CD95) expression in myeloid cells promotes obesity-induced muscle insulin resistance
Source: EMBO Mol Med. 2013 Nov 6;6(1):43–56. doi: 10.1002/emmm.201302962 (PMC3936487; doi:10.1002/emmm.201302962)
Supplement: Supplementary file 2 [file emmm0006-0043-sd2.pdf]

Supplemental Figure 1

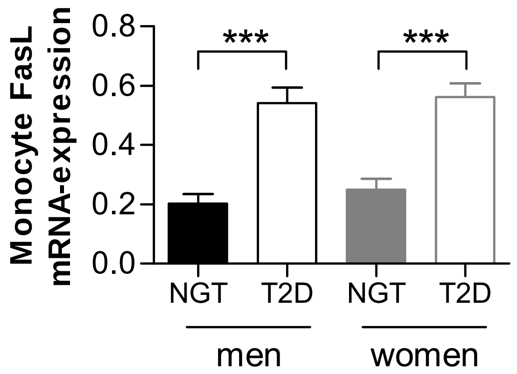

FasL expression in circulating human monocytes
Monocytes were isolated from whole human blood samples and FasL mRNA expression was measured normalized to HPRT. ***p < 0.0001 (Student's *t*-test). NGT: normal glucose tolerance (men n=61; women n=70); T2D: type 2 diabetes (men n=57; women n=58). Error bars represent SEM.
