# Supplementary material for: Fas (CD95) expression in myeloid cells promotes obesity-induced muscle insulin resistance
Source: EMBO Mol Med. 2013 Nov 6;6(1):43–56. doi: 10.1002/emmm.201302962 (PMC3936487; doi:10.1002/emmm.201302962)
Supplement: Supplementary file 3 [file emmm0006-0043-sd3.pdf]

Supplemental Figure 2

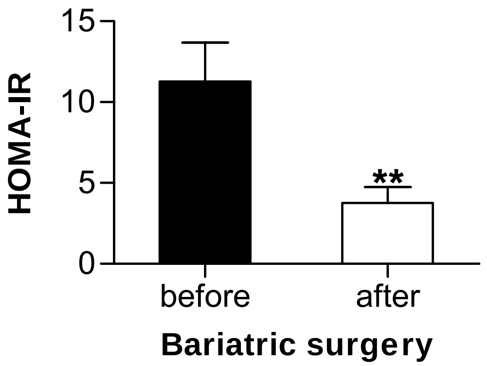

Improved insulin sensitivity HOMA-IR after bariatric surgery
HOMA-IR was determined in obese patients before and six months after bariatric surgery (gastric sleeve resection) (n=14), **p = 0.008 (Student's *t*-test). Error bars represent SEM.
