# Supplementary material for: Fas (CD95) expression in myeloid cells promotes obesity-induced muscle insulin resistance
Source: EMBO Mol Med. 2013 Nov 6;6(1):43–56. doi: 10.1002/emmm.201302962 (PMC3936487; doi:10.1002/emmm.201302962)
Supplement: Supplementary file 4 [file emmm0006-0043-sd4.pdf]

Supplemental Figure 3

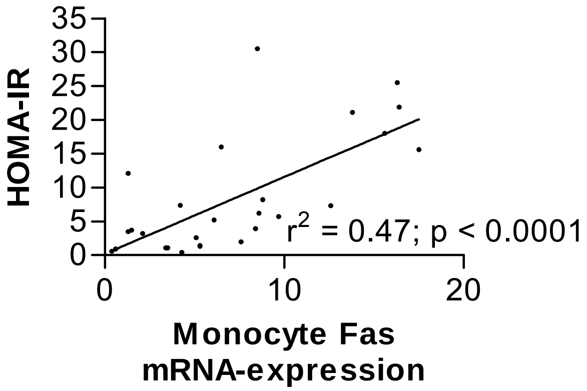

Positive correlation of monocytic Fas with HOMA-IR in the bariatric surgery group at baseline

Monocytes were isolated from whole human blood samples. Fas mRNA expression was measured, normalized to HPRT and correlated with HOMA-IR in the bariatric surgery group (baseline and after intervention; n=27).
