# Supplementary material for: Fas (CD95) expression in myeloid cells promotes obesity-induced muscle insulin resistance
Source: EMBO Mol Med. 2013 Nov 6;6(1):43–56. doi: 10.1002/emmm.201302962 (PMC3936487; doi:10.1002/emmm.201302962)
Supplement: Supplementary file 5 [file emmm0006-0043-sd5.pdf]

Supplemental Figure 4

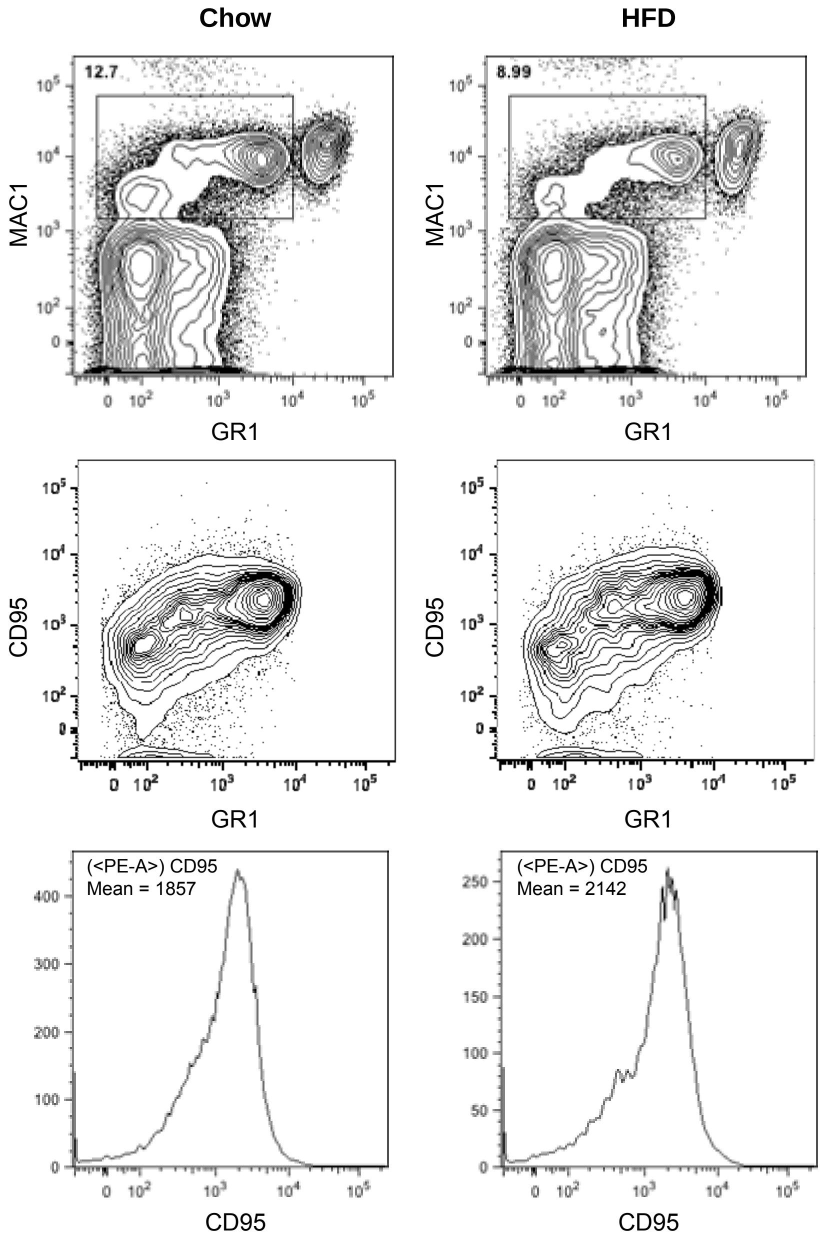

Flow cytometric analysis in chow- and HFD-fed C57BL6/J mice

Flow cytometric analysis of peripheral blood leukocytes of chow- and HFD-fed mice. Monocytes (GR1low/im MAC1+) were stained with respective antibodies and contour plots were gated on live cells. Inset is the percentage of cells within the GR1low/im Mac1+ gate.
