# Supplementary material for: Fas (CD95) expression in myeloid cells promotes obesity-induced muscle insulin resistance
Source: EMBO Mol Med. 2013 Nov 6;6(1):43–56. doi: 10.1002/emmm.201302962 (PMC3936487; doi:10.1002/emmm.201302962)
Supplement: Supplementary file 6 [file emmm0006-0043-sd6.pdf]

Supplemental Figure 5

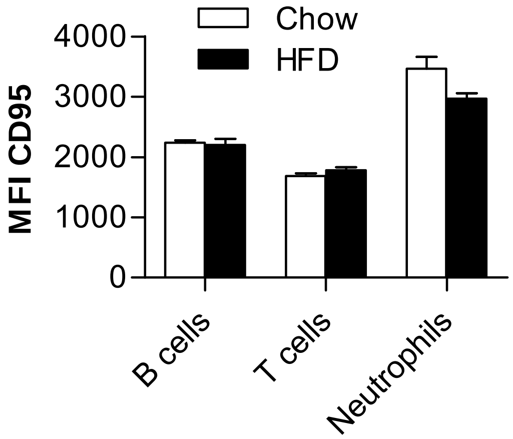

Flow cytometric analysis of peripheral blood leukocytes of chow- and HFD-fed mice. B-cell (B220+), T-cell (CD3e+) and neutrophils (GR1hi MAC1+) were stained with respective antibodies. Bar graphs show mean fluorescence intensity (MFI) of Fas (CD95) of live cells. n=6-8. Error bars represent SEM.
