# Supplementary material for: Fas (CD95) expression in myeloid cells promotes obesity-induced muscle insulin resistance
Source: EMBO Mol Med. 2013 Nov 6;6(1):43–56. doi: 10.1002/emmm.201302962 (PMC3936487; doi:10.1002/emmm.201302962)
Supplement: Supplementary file 7 [file emmm0006-0043-sd7.pdf]

# Supplemental Figure 6

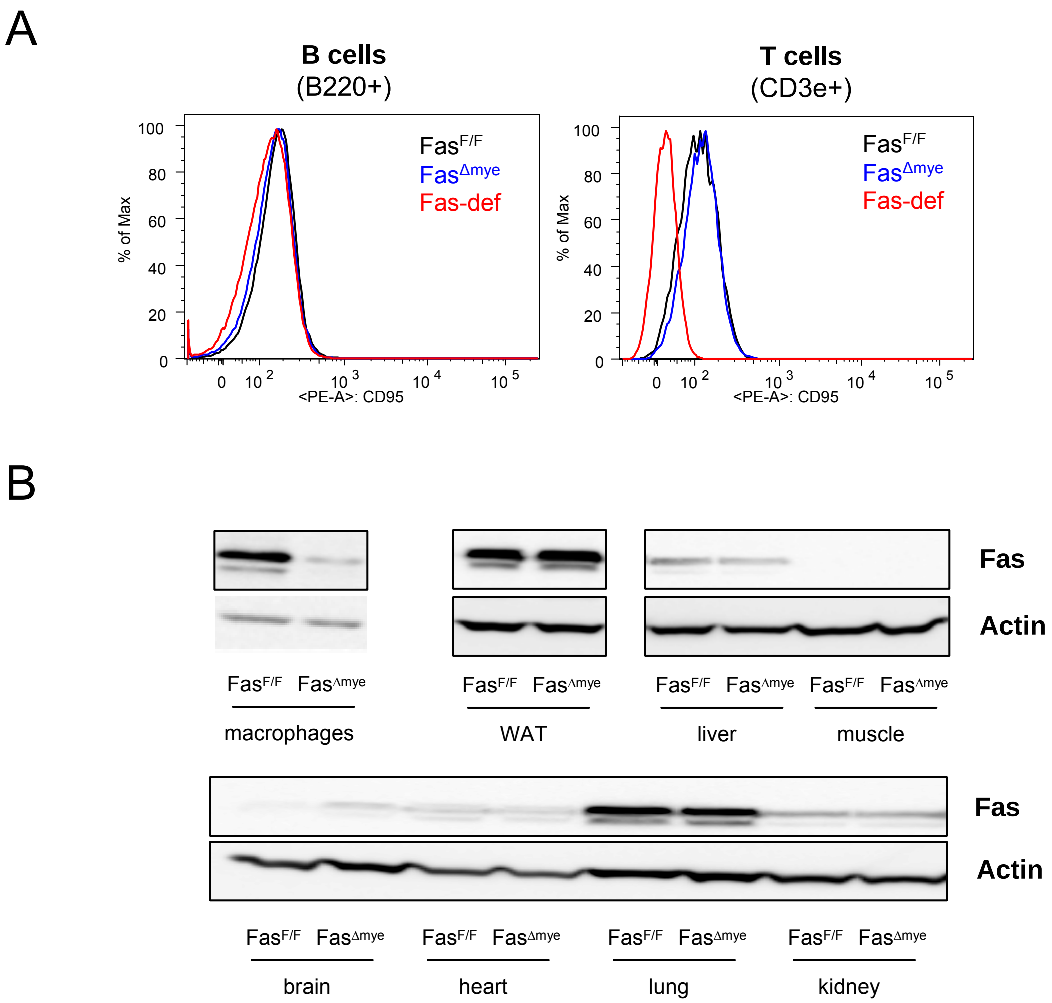


**Fas expression in Fas$^{F/F}$ and Fas$^{\Delta mye}$ mice**

(**A**) Flow cytometric analysis of peripheral blood leukocytes of Fas-deficient, Fas$^{F/F}$ and Fas$^{\Delta mye}$ mice. Cells were stained with respective antibodies and Fas fluorescence was measured. (**B**) Western blot analysis of Fas protein levels in respective tissues of Fas$^{F/F}$ and Fas$^{\Delta mye}$ mice.
