# Supplementary material for: Fas (CD95) expression in myeloid cells promotes obesity-induced muscle insulin resistance
Source: EMBO Mol Med. 2013 Nov 6;6(1):43–56. doi: 10.1002/emmm.201302962 (PMC3936487; doi:10.1002/emmm.201302962)
Supplement: Supplementary file 8 [file emmm0006-0043-sd8.pdf]

Supplemental Figure 7

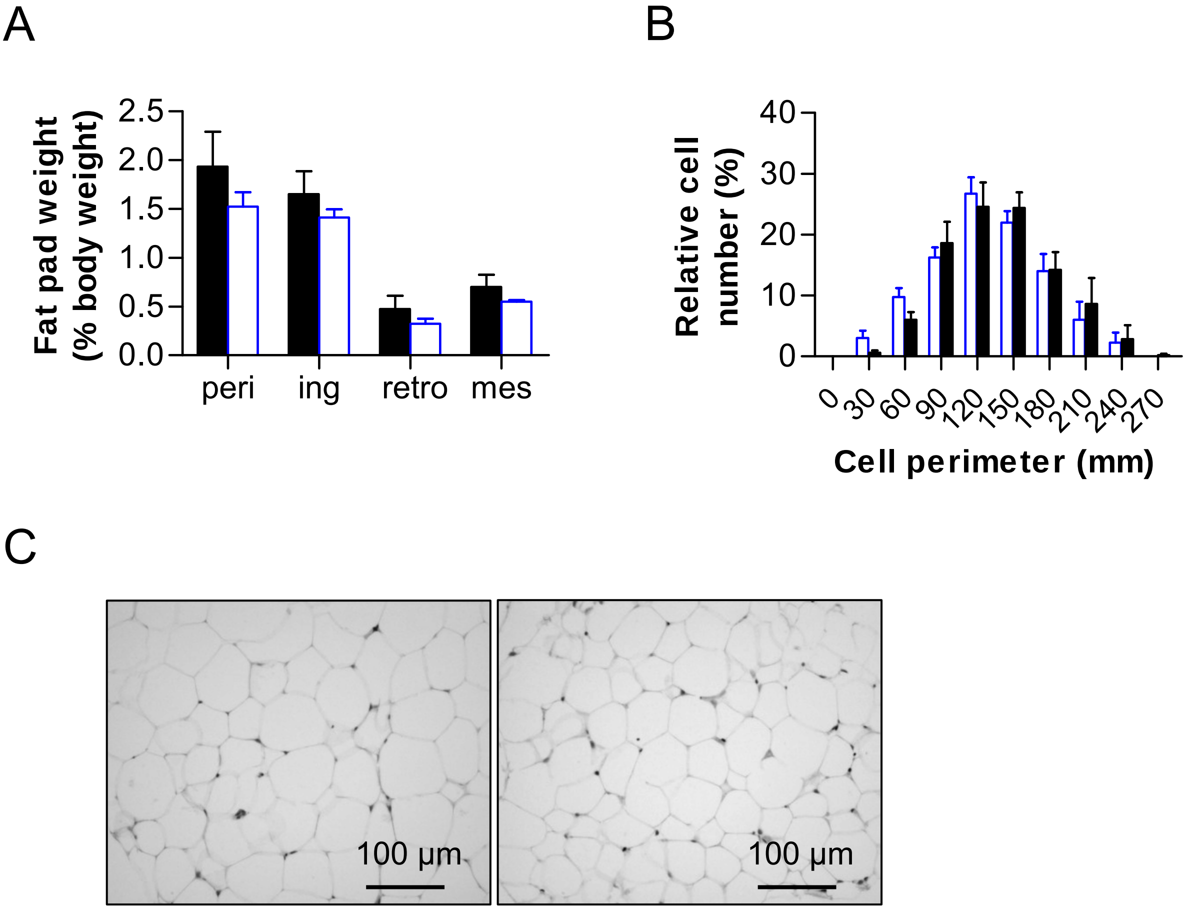

Fas$^{F/F}$ Fas$^{\Delta mye}$

Adipose tissue characteristics of Fas$^{F/F}$ and Fas$^{\Delta mye}$ mice

(**A**) Fat pad weights of HFD-fed Fas$^{F/F}$ (black bars) and Fas$^{\Delta mye}$ (blue bars) mice. n=6-7. (**B**) Adipocyte perimeters of HFD-fed Fas$^{F/F}$ and Fas$^{\Delta mye}$ mice. n=4-5. (**C**) Representative hematoxylin- and eosin-stained histological sections of white adipose tissue of HFD-fed Fas$^{F/F}$ and Fas$^{\Delta mye}$ mice. Error bars represent SEM.
