# Supplementary material for: Fas (CD95) expression in myeloid cells promotes obesity-induced muscle insulin resistance
Source: EMBO Mol Med. 2013 Nov 6;6(1):43–56. doi: 10.1002/emmm.201302962 (PMC3936487; doi:10.1002/emmm.201302962)
Supplement: Supplementary file 9 [file emmm0006-0043-sd9.pdf]

Supplemental Figure 8

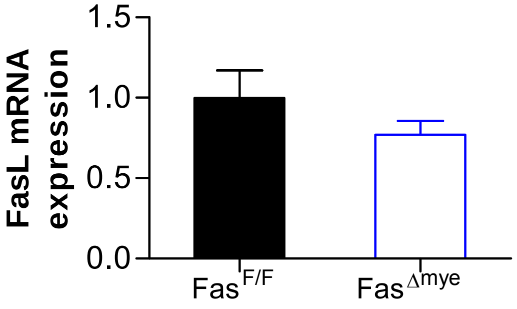

Similar FasL expression in blood leukocytes of HFD-fed mice

Leukocyte-mRNA expression of FasL in HFD-fed $Fas^{F/F}$ and $Fas^{\Delta mye}$ mice. n=3-5. Error bars represent SEM.
