# Supplementary material for: Fas (CD95) expression in myeloid cells promotes obesity-induced muscle insulin resistance
Source: EMBO Mol Med. 2013 Nov 6;6(1):43–56. doi: 10.1002/emmm.201302962 (PMC3936487; doi:10.1002/emmm.201302962)
Supplement: Supplementary file 10 [file emmm0006-0043-sd10.pdf]

Supplemental Figure 9

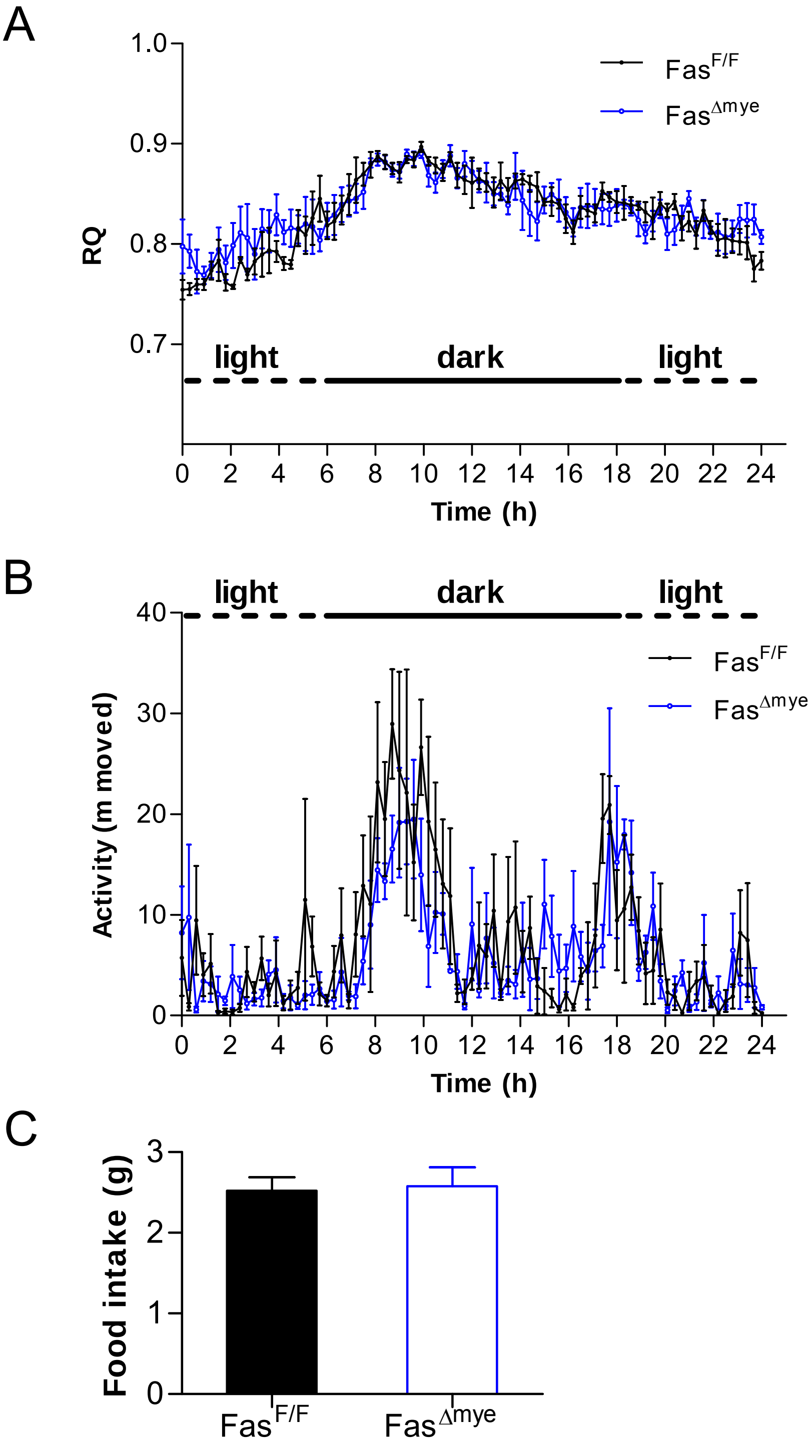

Similar respiratory quotient, locomotor activity and food intake in Fas$^{F/F}$ and Fas$^{\Delta mye}$ mice

(**A** and **B**) Respiratory quotient (RQ) and activity was determined in metabolic cages in HFD-fed Fas$^{F/F}$ and Fas$^{\Delta mye}$ mice. n=4. (**C**) Food intake was measured in metabolic cages in Fas$^{F/F}$ (black bar) and Fas$^{\Delta mye}$ (blue bar) mice. n=3-4. Error bars represent SEM.
