# Supplementary material for: Fas (CD95) expression in myeloid cells promotes obesity-induced muscle insulin resistance
Source: EMBO Mol Med. 2013 Nov 6;6(1):43–56. doi: 10.1002/emmm.201302962 (PMC3936487; doi:10.1002/emmm.201302962)
Supplement: Supplementary file 11 [file emmm0006-0043-sd11.pdf]

Supplemental Figure 10

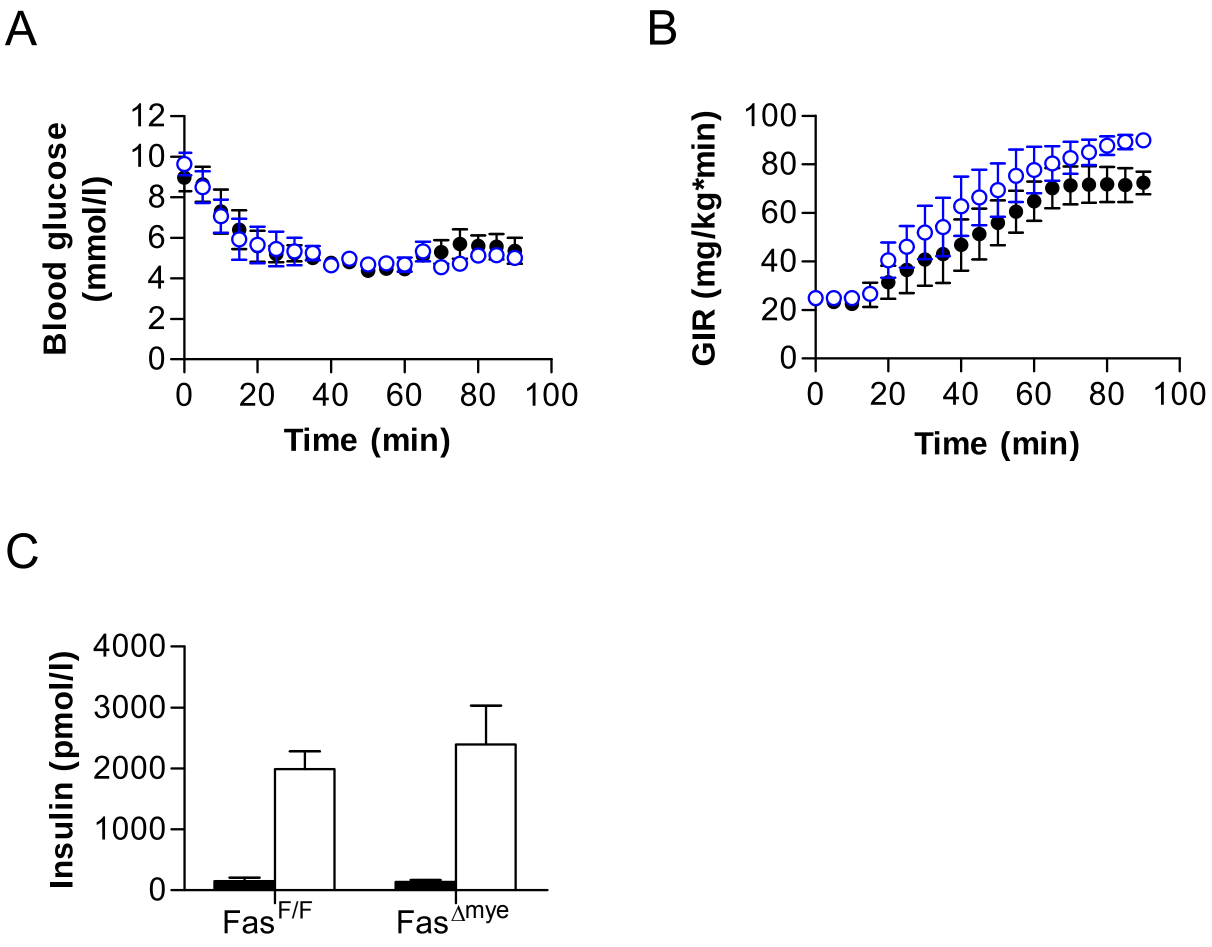

Blood glucose concentrations, glucose infusion rates and plasma insulin levels during hyperinsulinemic-euglycemic clamp

(**A**) Blood glucose levels were clamped upon insulin infusion at about 5 mmol/l in HFD-fed $Fas^{F/F}$ (black circles) and $Fas^{\Delta mye}$ (blue circles) mice. n=4-5. (**B**) In order to maintain euglycemia, glucose infusion rate was adjusted over time in $Fas^{F/F}$ (black circles) and $Fas^{\Delta mye}$ (blue circles) mice. n=4-5. (**C**) Plasma insulin levels in the basal state (black bars) and after insulin infusion (white bars). n=4-5. Error bars represent SEM.
