# Supplementary material for: Fas (CD95) expression in myeloid cells promotes obesity-induced muscle insulin resistance
Source: EMBO Mol Med. 2013 Nov 6;6(1):43–56. doi: 10.1002/emmm.201302962 (PMC3936487; doi:10.1002/emmm.201302962)
Supplement: Supplementary file 12 [file emmm0006-0043-sd12.pdf]

Supplemental Figure 11

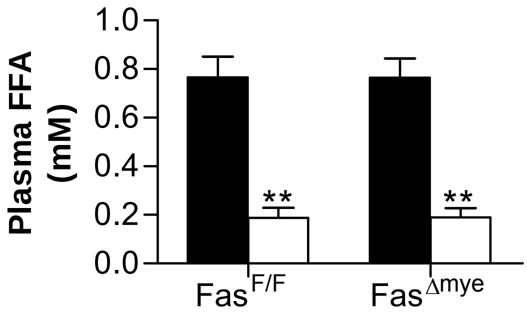

Similar plasma FFA levels during hyperinsulinemic-euglycemic clamp
Plasma FFA levels in the basal state (black bars) and after insulin infusion (white bars) in HFD-fed $Fas^{F/F}$ and $Fas^{\Delta mye}$ mice. n=4-5. **p = 0.003 ($Fas^{F/F}$) and **p = 0.006 ($Fas^{\Delta mye}$) (Student's *t*-test). Error bars represent SEM.
