# Supplementary material for: Fas (CD95) expression in myeloid cells promotes obesity-induced muscle insulin resistance
Source: EMBO Mol Med. 2013 Nov 6;6(1):43–56. doi: 10.1002/emmm.201302962 (PMC3936487; doi:10.1002/emmm.201302962)
Supplement: Supplementary file 13 [file emmm0006-0043-sd13.pdf]

Supplemental Figure 12

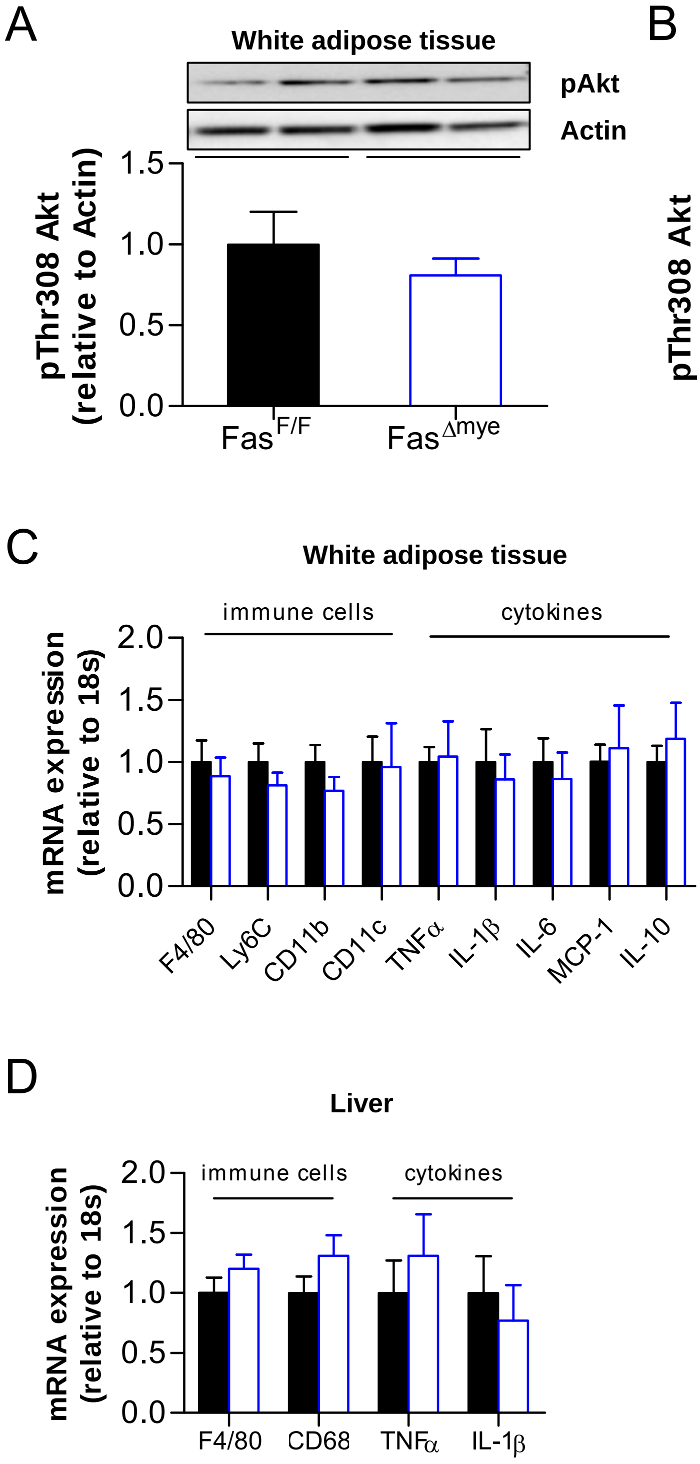

Improved insulin sensitivity HOMA-IR after bariatric surgery
HOMA-IR was determined in obese patients before and six months after bariatric surgery (gastric sleeve resection) (n=14), **p < 0.01 (Student's *t*-test). Error bars represent SEM.

E

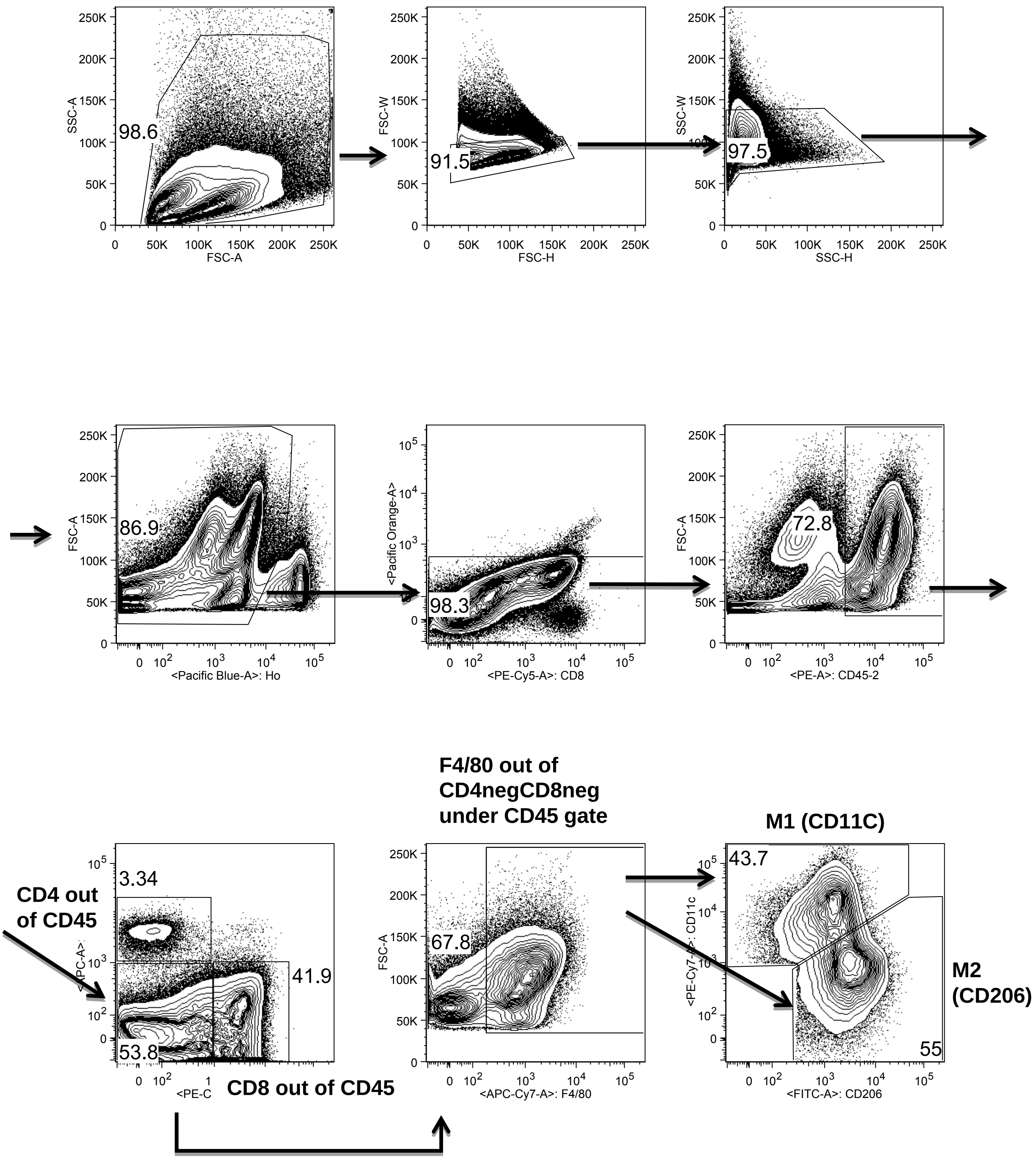

98.6
91.5
97.5
86.9
98.3
72.8
F4/80 out of
CD4negCD8neg
under CD45 gate
M1 (CD11C)
CD4 out
of CD45
3.34
41.9
53.8
CD8 out of CD45
67.8
43.7
M2
(CD206)
55
FSC-A
SSC-A
FSC-H
FSC-W
SSC-H
SSC-W
<Pacific Blue-A>: Ho
<Pacific Orange-A>
<PE-Cy5-A>: CD8
<PE-A>: CD45-2
<APC-Cy7-A>: F4/80
<FITC-A>: CD206

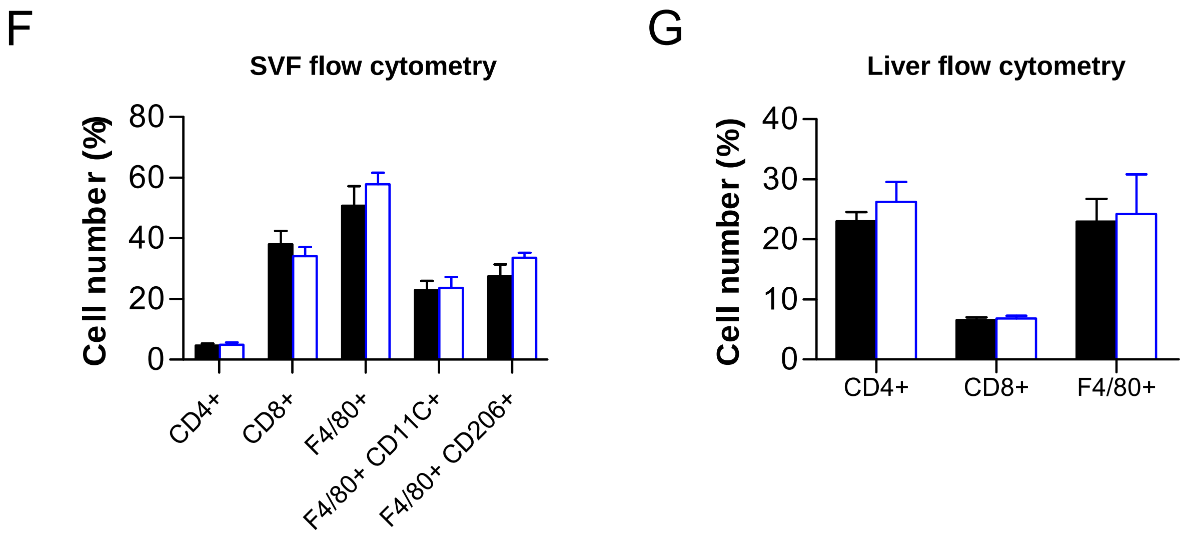

Similar immune cell infiltration in white adipose tissue and livers of Fas$^{F/F}$ and Fas$^{\Delta mye}$ mice

(**A** and **B**) Representative Western blots of total WAT and liver lysates of HFD-fed Fas$^{F/F}$ and Fas$^{\Delta mye}$ mice. Results are the means ± SEM of 4-5 mice. (**C** and **D**) mRNA expression of respective genes in WAT and livers of Fas$^{F/F}$ (black bars) and Fas$^{\Delta mye}$ (blue bars) mice. n=4-5. (**E**) Gating strategy of flow cytometric analysis. (**F** and **G**) Flow cytometric analysis of stromal vascular fraction (SVF) of white adipose tissue and liver cells of HFD-fed Fas$^{F/F}$ (black bars) and Fas$^{\Delta mye}$ (blue bars) mice. Cells were stained with respective antibodies and fluorescence was measured. n=5. All error bars represent SEM.
