# Supplementary material for: Fas (CD95) expression in myeloid cells promotes obesity-induced muscle insulin resistance
Source: EMBO Mol Med. 2013 Nov 6;6(1):43–56. doi: 10.1002/emmm.201302962 (PMC3936487; doi:10.1002/emmm.201302962)
Supplement: Supplementary file 14 [file emmm0006-0043-sd14.pdf]

Supplemental Figure 13

A

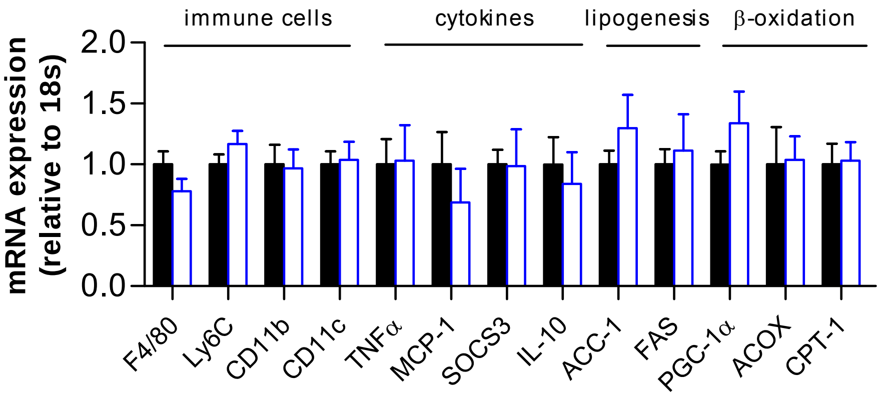

B

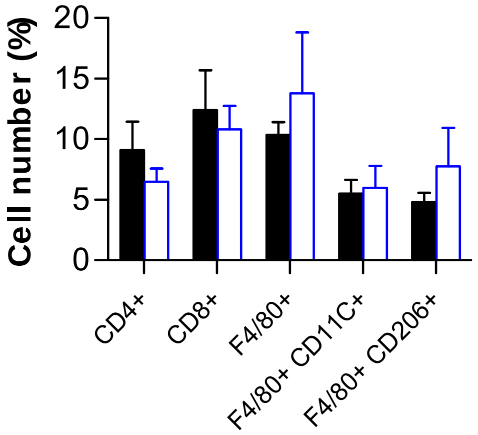

mRNA expression and flow cytometric analysis of skeletal muscle from $Fas^{F/F}$ and $Fas^{\Delta mye}$ mice

(**A**) mRNA expression of respective genes in skeletal muscle of HFD-fed $Fas^{F/F}$ (black bars) and $Fas^{\Delta mye}$ (blue bars) mice. n=6-10. (**B**) Flow cytometric analysis of skeletal muscle cells of $Fas^{F/F}$ (black bars) and $Fas^{\Delta mye}$ (blue bars) mice. Cells were stained with respective antibodies and fluorescence was measured. n=5. All error bars represent SEM.
