# Supplementary material for: Fas (CD95) expression in myeloid cells promotes obesity-induced muscle insulin resistance
Source: EMBO Mol Med. 2013 Nov 6;6(1):43–56. doi: 10.1002/emmm.201302962 (PMC3936487; doi:10.1002/emmm.201302962)
Supplement: Supplementary file 15 [file emmm0006-0043-sd15.pdf]

Supplemental Figure 14

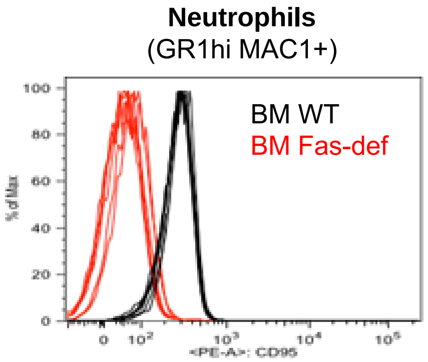

Reduced Fas protein content in myeloid cells of BM Fas-def compared to BM WT mice

Flow cytometric analysis of peripheral blood leukocytes of BM WT and BM Fas-def mice. Neutrophils were stained with respective antibodies and Fas fluorescence was measured in respective fraction. n=6-7.
