# Supplementary material for: Fas (CD95) expression in myeloid cells promotes obesity-induced muscle insulin resistance
Source: EMBO Mol Med. 2013 Nov 6;6(1):43–56. doi: 10.1002/emmm.201302962 (PMC3936487; doi:10.1002/emmm.201302962)
Supplement: Supplementary file 16 [file emmm0006-0043-sd16.pdf]

Supplemental Figure 15

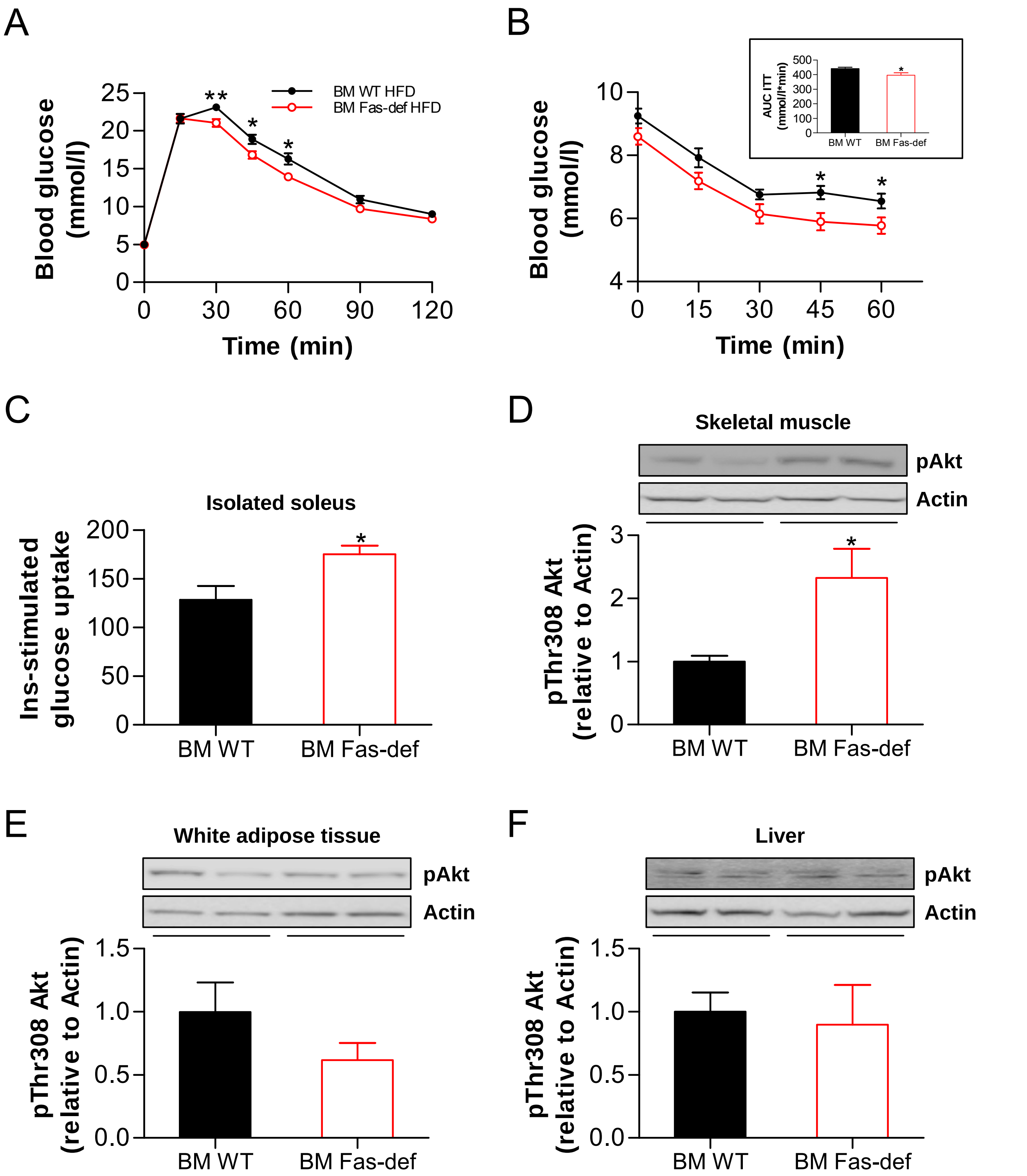

Improved skeletal muscle insulin sensitivity in HFD-fed BM Fas-def compared to BM WT mice

(**A**) Intra-peritoneal glucose-tolerance test in HFD-fed BM WT and BM Fas-def mice. n=11-12, *p = 0.017 and 0.013 (after 45 min and 60 min, respectively), **p = 0.005 (Student's *t*-test). Error bars represent SEM. (**B**) Intra-peritoneal insulin-tolerance test. n=9-12. Inserted

graphs depict the respective analysis of the area under the curve. *p = 0.014, 0.040 and 0.021 (after 45 min, 60 min and for area under the curve, respectively) (Student's *t*-test). (**C**) Insulin-stimulated glucose uptake in isolated soleus muscle relative to basal uptake. n=4-5, *p = 0.036 (Student's *t*-test). (**D** to **F**) Representative Western blots of total lysates of BM WT and BM Fas-def mice. Graphs show results of 3-4 mice. *p = 0.022 (Student's *t*-test). All error bars represent SEM.
