# Supplementary material for: Fas (CD95) expression in myeloid cells promotes obesity-induced muscle insulin resistance
Source: EMBO Mol Med. 2013 Nov 6;6(1):43–56. doi: 10.1002/emmm.201302962 (PMC3936487; doi:10.1002/emmm.201302962)
Supplement: Supplementary file 17 [file emmm0006-0043-sd17.pdf]

Supplemental Figure 16

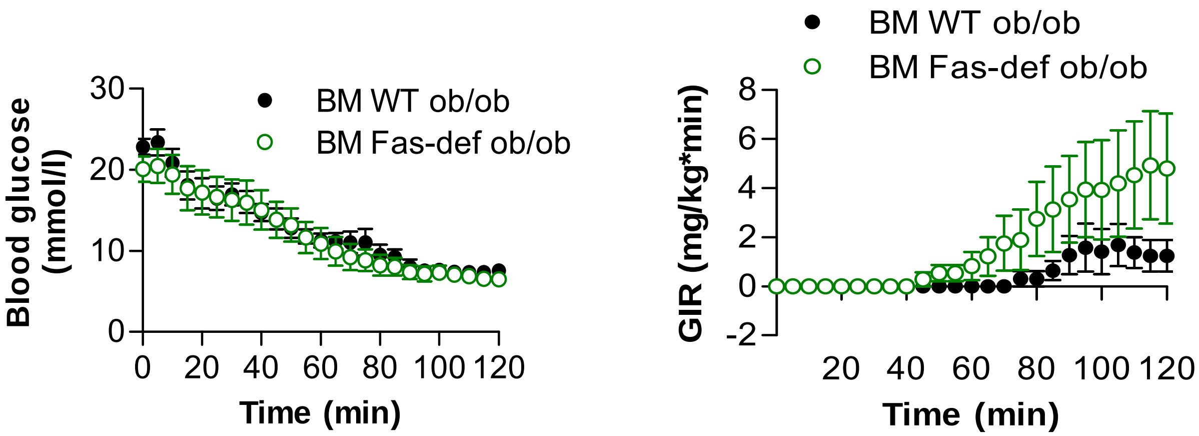

Blood glucose concentrations and glucose infusion rates during hyperinsulinemic-euglycemic clamp

(**A**) Blood glucose levels were clamped upon insulin infusion at about 7-8 mmol/l in BM WT ob/ob (black circles) and BM Fas-def ob/ob (green circles) mice. (**B**) In order to maintain euglycemia, glucose infusion rate was adjusted over time in BM WT ob/ob (black circles) and BM Fas-def ob/ob (green circles) mice. n=5-6. Error bars represent SEM.
