# Supplementary material for: Fas (CD95) expression in myeloid cells promotes obesity-induced muscle insulin resistance
Source: EMBO Mol Med. 2013 Nov 6;6(1):43–56. doi: 10.1002/emmm.201302962 (PMC3936487; doi:10.1002/emmm.201302962)
Supplement: Supplementary file 18 [file emmm0006-0043-sd18.pdf]

Supplemental Figure 17

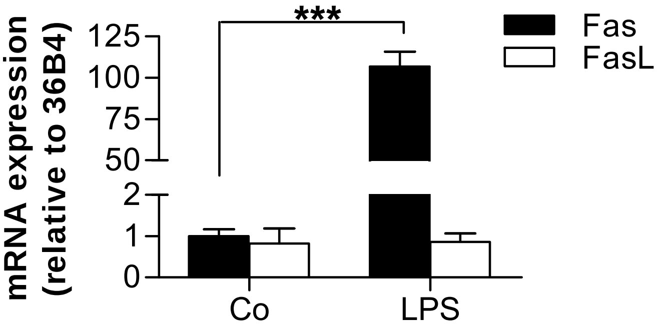

LPS does not increase FasL expression in RAW cells
mRNA expression of Fas and FasL in RAW cells treated with or without LPS (100 ng/ml for 6 hours). n=3. ***p = 0.0003 (Student's *t*-test). Error bars represent SEM.
