# Supplementary material for: Fas (CD95) expression in myeloid cells promotes obesity-induced muscle insulin resistance
Source: EMBO Mol Med. 2013 Nov 6;6(1):43–56. doi: 10.1002/emmm.201302962 (PMC3936487; doi:10.1002/emmm.201302962)
Supplement: Supplementary file 19 [file emmm0006-0043-sd19.pdf]

Supplemental Figure 18

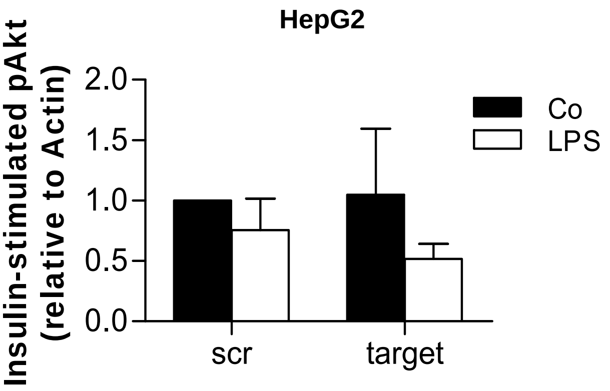

No effect of Fas down-regulation in RAW cells on conditioned medium-induced insulin resistance in HepG2 cells

HepG2 cells were incubated overnight with conditioned media and then stimulated with 100 nM insulin for 10 min. Phosphorylation of Akt on Thr308 was detected by Western blot technique. n=3. Error bars represent SEM.
