# Supplementary material for: Fas (CD95) expression in myeloid cells promotes obesity-induced muscle insulin resistance
Source: EMBO Mol Med. 2013 Nov 6;6(1):43–56. doi: 10.1002/emmm.201302962 (PMC3936487; doi:10.1002/emmm.201302962)
Supplement: Supplementary file 20 [file emmm0006-0043-sd20.pdf]

Supplemental Figure 19

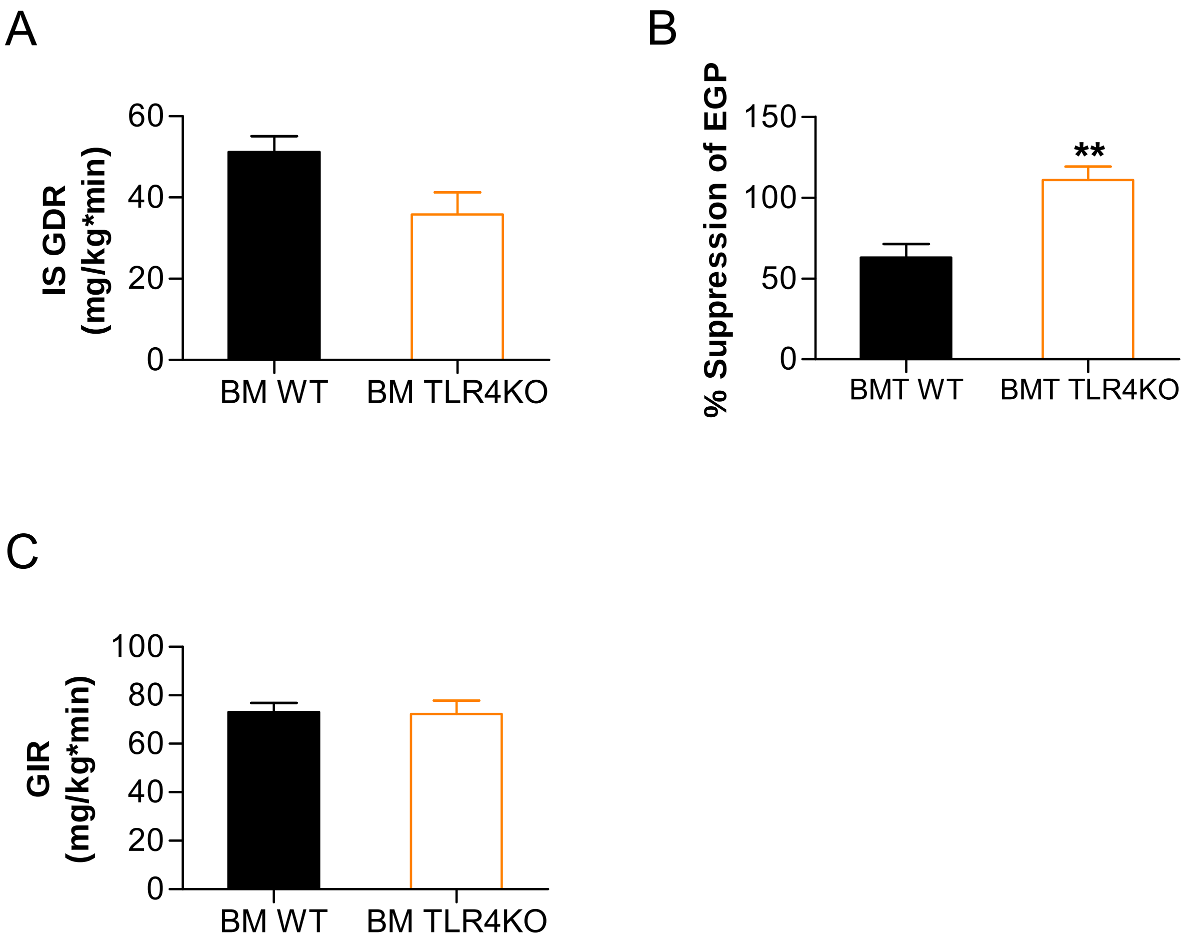

Hyperinsulinemic-euglycemic clamp in myeloid-specific TLR4 KO mice

Hyperinsulinemic-euglycemic clamp studies with insulin-stimulated glucose disposal rate (IS GDR) (**A**), suppression of endogenous glucose production (EGP) (**B**) and glucose infusion rate (GIR) (**C**) were performed in BM WT and BM TLR4KO mice. n=6, **p = 0.0022 (Student's *t*-test). All error bars represent SEM.
