# Supplementary material for: Fas (CD95) expression in myeloid cells promotes obesity-induced muscle insulin resistance
Source: EMBO Mol Med. 2013 Nov 6;6(1):43–56. doi: 10.1002/emmm.201302962 (PMC3936487; doi:10.1002/emmm.201302962)
Supplement: Supplementary file 21 [file emmm0006-0043-sd21.pdf]

Supplemental Table 1 Phenotypic characteristics of HFD-fed BMT mice

	BM WT	BM Fas-def
Body weight (g)	26.8 ± 0.5	26.1 ± 0.4
Blood glucose (mmol/l)	10.1 ± 0.4	8.6 ± 0.4*
Insulin (pmol/l)	170.5 ± 26.5	148.3 ± 13.4
FFA (mmol/l)	1.06 ± 0.04	1.13 ± 0.09
TG (mg/dl)	186.0 ± 10.1	158.4 ± 8.0
Adiponectin (μg/ml)	52.8 ± 2.6	51.5 ± 2.0
Leptin (pg/ml)	81.1 ± 8.3	95.2 ± 8.9
TNFα (pg/ml)	3.6 ± 0.5	2.1 ± 0.2*
IL-6 (pg/ml)	2.0 ± 0.5	1.5 ± 0.2
MCP-1 (pg/ml)	6.8 ± 0.6	6.3 ± 0.5
IL-10 (pg/ml)	n.d.	n.d.

Mice were fasted for 8 hours. Results are the means ± SEM of 7 to 8 mice. *$p < 0.05$ (Student's *t* test). n.d. = not detectable.
