# Supplementary material for: Fas (CD95) expression in myeloid cells promotes obesity-induced muscle insulin resistance
Source: EMBO Mol Med. 2013 Nov 6;6(1):43–56. doi: 10.1002/emmm.201302962 (PMC3936487; doi:10.1002/emmm.201302962)
Supplement: Supplementary file 22 [file emmm0006-0043-sd22.pdf]

Supplemental Table 2 Multivariate linear regression models

		monocyte Fas mRNA	
		Beta	p-value
Model 1	Age	0.195	*0.001*
	Gender	-0.033	0.580
	BMI	0.336	*<0.001*
Model 2	Age	0.134	*0.020*
	Gender	-0.027	0.634
	BMI	0.192	*0.002*
	LPS	**0.338**	*<0.001*
Model 3	Age	0.163	*0.017*
	Gender	-0.018	0.722
	BMI	0.271	*0.001*
	TNFα	**0.205**	*<0.01*
Model 4	Age	0.227	*0.001*
	Gender	0.027	0.702
	GDR	**-0.511**	*<0.001*
Model 5	Age	0.231	*0.001*
	Gender	0.029	0.675
	BMI	0.063	0.413
	GDR	**-0.484**	*<0.001*
